# Tau- and α-synuclein-targeted gold nanoparticles: applications, opportunities, and future outlooks in the diagnosis and therapy of neurodegenerative diseases

**DOI:** 10.1186/s12951-024-02526-0

**Published:** 2024-05-13

**Authors:** Andreas Tapia-Arellano, Pablo Cabrera, Elizabeth Cortés-Adasme, Ana Riveros, Natalia Hassan, Marcelo J. Kogan

**Affiliations:** 1 Instituto Universitario de Investigación y Desarrollo Tecnológico (IDT), Universidad Tecnológica Metropolitana, Santiago, Chile; 2 Facultad de Cs. Qcas. y Farmacéuticas, Universidad de Chile, Santiago, Chile; 3 Advanced Center for Chronic Diseases (ACCDis), Santiago, Chile; 4 Millenium Nucleus in NanoBioPhysics, Valparaíso, Chile

**Keywords:** Alzheimer's disease, Parkinson’s disease, Protein misfolding, Diagnosis, Therapy, Theragnosis

## Abstract

The use of nanomaterials in medicine offers multiple opportunities to address neurodegenerative disorders such as Alzheimer's and Parkinson's disease. These diseases are a significant burden for society and the health system, affecting millions of people worldwide without sensitive and selective diagnostic methodologies or effective treatments to stop their progression. In this sense, the use of gold nanoparticles is a promising tool due to their unique properties at the nanometric level. They can be functionalized with specific molecules to selectively target pathological proteins such as Tau and α-synuclein for Alzheimer’s and Parkinson’s disease, respectively. Additionally, these proteins are used as diagnostic biomarkers, wherein gold nanoparticles play a key role in enhancing their signal, even at the low concentrations present in biological samples such as blood or cerebrospinal fluid, thus enabling an early and accurate diagnosis. On the other hand, gold nanoparticles act as drug delivery platforms, bringing therapeutic agents directly into the brain, improving treatment efficiency and precision, and reducing side effects in healthy tissues. However, despite the exciting potential of gold nanoparticles, it is crucial to address the challenges and issues associated with their use in the medical field before they can be widely applied in clinical settings. It is critical to ensure the safety and biocompatibility of these nanomaterials in the context of the central nervous system. Therefore, rigorous preclinical and clinical studies are needed to assess the efficacy and feasibility of these strategies in patients. Since there is scarce and sometimes contradictory literature about their use in this context, the main aim of this review is to discuss and analyze the current state-of-the-art of gold nanoparticles in relation to delivery, diagnosis, and therapy for Alzheimer’s and Parkinson’s disease, as well as recent research about their use in preclinical, clinical, and emerging research areas.

**Graphical Abstract:**

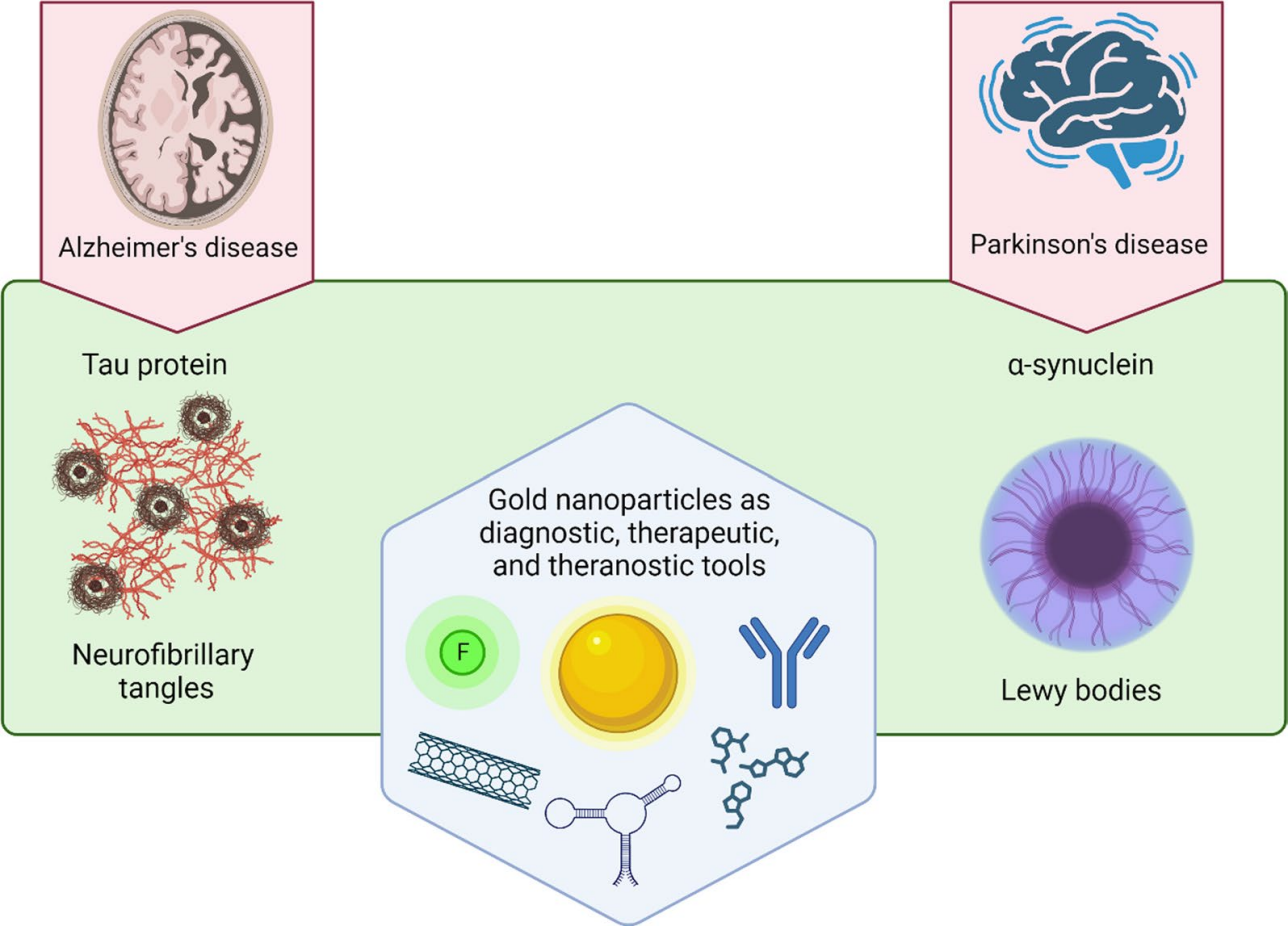

## Introduction

Neurodegenerative diseases have become emergent and prevalent illnesses with age, being Alzheimer’s disease (AD) and Parkinson’s disease (PD), two of the most extended disorders [1, 2]. Even though these neurodegenerative diseases have different cognitive disabilities, and clinical manifestations [3], both share a common molecular mechanism: misfolding proteins that underlie the progressive and chronic nature of each disease [4, 5]. Alzheimer´s disease (AD) is the most common kind of dementia [6–8], accompanied by memory loss and dependence on personal daily activities. It is characterized by the presence of two misfolding proteins: amyloid-β peptide (Aβ) and Tau protein, which form highly insoluble aggregates. In this regard, the Aβ peptide aggregates extracellularly, causing synaptic damage, activation of proinflammatory pathways, mitochondrial dysfunction, oxidative stress damage, and neuronal death, promoting the neurodegenerative process and cognitive impairment [9–13].

For over 30 years, Aβ has been considered the mainstream that underlies and dominate the AD research, with extensive literature and clinical trials at the expense of Tau protein [14, 15]. Recent studies have shown a preponderant role of Tau protein in the propagation of AD, playing a key interplay with Aβ in the neurodegenerative effect [16–19]. In fact, there are more evidence that show the key role of Tau as an active agent, refuting the Amyloid cascade as the main and unique actor of neurodegeneration[15, 20], and turning the focus on AD research field. In this sense, Tau protein is a soluble protein predominantly found in neuronal axons and associated with microtubules, playing a fundamental role in microtubule stability under physiological conditions, being relevant from a structural and axonal traffic point of view in neurons [21]. The loss of Tau protein function has been associated not only with AD, but also with various tauopathies: Pick disease, progressive supranuclear palsy, and corticobasal degeneration [22]. The human tau gene is localized in chromosome 17 and expresses six isoforms of 351–441 amino acid residues with a molecular weight of 45–65 kDa [23], being the called 4-R (4R) and 3-R (3R) isoforms mainly located in the axons of an adult brain. Under pathological conditions, 4R and 3R Tau are found to be hyperphosphorylated at threonine and serine residues [23], producing structural instability and disruptions in neuronal transport processes [24]. This hyperphosphorylation could also promote an aggregation process forming the neurofibrillary tangles (NFTs) [25]. Additionally, different intermediate and highly toxic oligomeric species are produced throughout this aggregation process, triggering inflammatory processes and oxidative stress, which ultimately lead to neuronal death [24, 26]. Thus, total Tau (T-tau), hyperphosphorylated Tau (P-Tau), and the oligomeric species have been used as key biomarkers for AD [27] even though they are found at low levels, making their detection difficult in biological fluids such as cerebrospinal fluid (CSF) (low 195 pg/mL or 4.7 pM) [28].

On the other hand, PD is characterized by motor impairments such as uncontrollable movements and difficulty with balance and coordination [5]. At the molecular level, there are intracellular lesions called Lewy bodies, which are composed of aggregates of α-synuclein (α-syn) protein [29]. This protein is part of the synuclein family which consists of three members: α-, β-, and γ-synuclein, that range from 127 to 140 residues [30]. However, only α-syn is present in the Lewy bodies and, therefore, possesses an attractive clinical perspective. There is no evidence that β- and γ-synuclein are present in the Lewy bodies because they have been demonstrated to fail to assemble into filaments, remaining in a random coil conformation [31]. α-syn is located in the neuronal presynaptic terminals. It is a small acidic protein of 14 kDa that is found as a monomer in the cytosol or as an α-helix structure in association with phospholipids under physiological conditions. The α-syn protein consists of three different parts: an amphiphilic N-terminal (residues 1–60), a hydrophobic region called NAC (non-amyloid-β component, residues 61–95), and an acidic C-terminal (residues 96–140) [32]. In this regard, the NAC region is highlighted because it plays an essential role in assembling monomeric α-syn into β-sheet-rich aggregates [33]. Under pathological conditions, monomers of α-syn exhibit a similar aggregation behavior as Tau, forming various species, including dimers, oligomers, protofibrils, and finally, fibrils, which eventually constitute the Lewy bodies [29]. Likewise, α-syn oligomers may drive the primary neurotoxicity, playing a critical role in neurodegeneration [34, 35]. Even though α-syn oligomers have emerged as potential biomarkers, the current methods have encountered challenges in accurately discriminating their morphology and size to detect them at appropriate concentrations, thus hindering their analysis and study [36, 37]. Finally, α-syn can also suffer genetic mutations and post-translational modifications such as phosphorylation, ubiquitination, and oxidation. These modifications have been observed to promote the misfolding process and correlate with disease progression [38] and, therefore, are being utilized as interesting biomarkers to follow the disease.

Even though the amino acid sequences of Tau and α-syn proteins are different, they both accumulate in pathological conditions, go through the same aggregation process, and generate toxic oligomers involved in the onset of AD and PD, respectively. In this sense, these oligomers possess a transient nature, low concentration in biological samples, and a wide range of different sizes and morphologies, making their analysis, quantification, and detection a remaining challenge in their use as promising biomarkers [37, 39]. Therefore, it is mandatory to develop a strategy that detects these species to generate future and potential therapies for AD and PD. Additionally, other species of Tau and α-syn may be used as targets to generate specific biomarkers to discriminate pathological concentrations in biological samples or develop therapies to modify the underlying aggregation processes [40–42]. Nevertheless, each of these approaches is hindered by factors that prevent the achievement of an effective treatment or diagnosis [43, 44], including side effects, the blood–brain barrier (BBB), and the challenges associated with the specificity and sensitivity of the new strategies [45, 46]. Overcoming these difficulties is crucial for developing new therapies or early diagnosis.

**Fig. 1:**
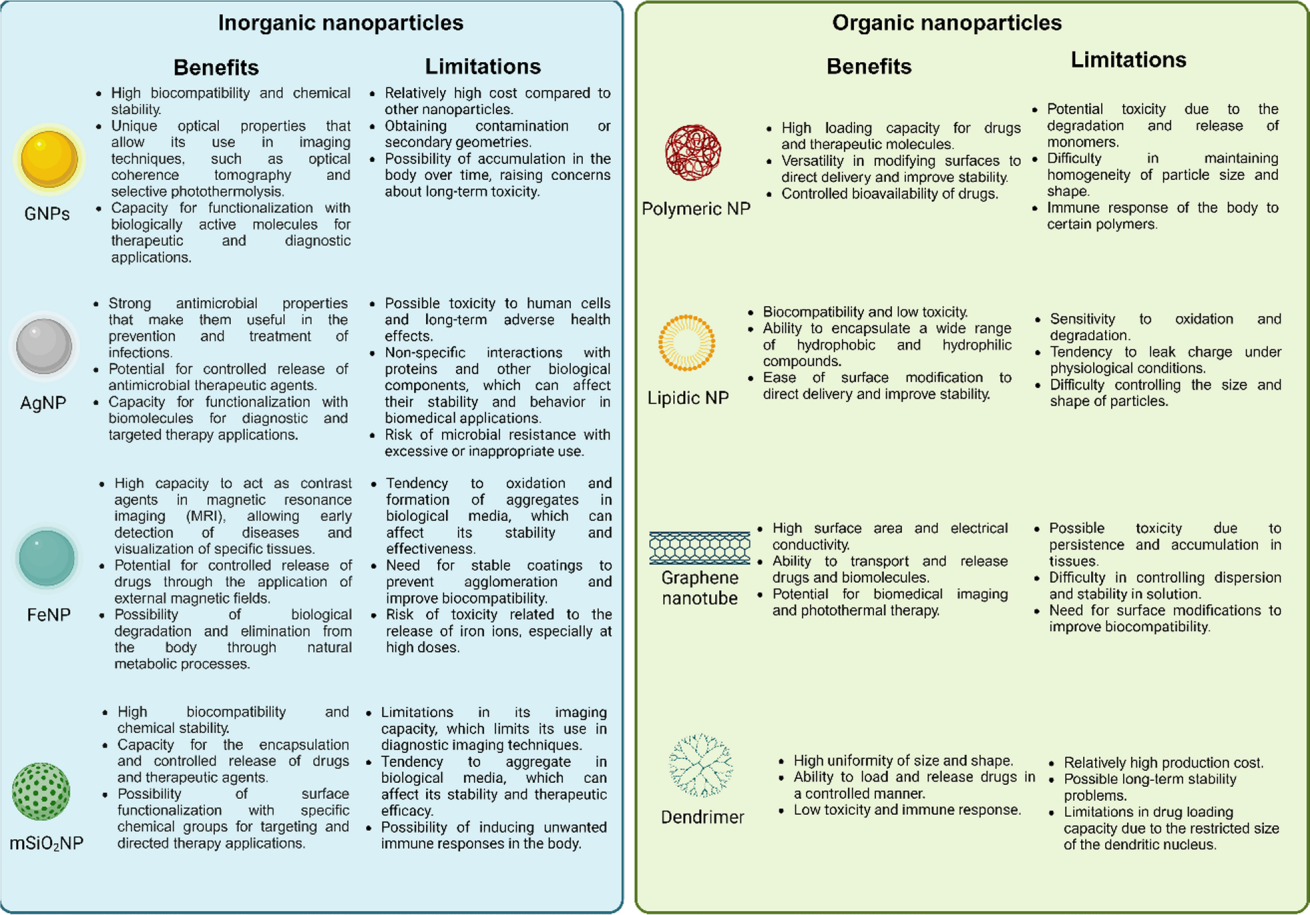
Comparison between the main inorganic and organic nanoparticles composed by different materials. Their benefits and limitations are highlighted to guide their use and potential applications. Created with BioRender.com

Nowadays, nanomaterials have acquired a high level of relevance in the nanobiotechnology field, and specifically in biomedicine, due to their several advantages, such as nanometer scale, relative low toxicity, and the capability of crossing biological membranes (including the BBB) [47]. Among these, gold nanoparticles (GNP) are the most studied and exhibit versatile features such as their high compatibility and stability in relevant biological mediums, tunable shape, and high surface area to be functionalized with different molecules over other kinds of nanomaterials [48–50]. In this regard, GNP are easily functionalized, becoming them drug carriers to target specific organs [51, 52] or specific molecules [53], enhancing their delivery capabilities compared to other materials (Fig. 1) [54]. Indeed, GNP possess unique properties to increase or amplify signals over other nanomaterials[55, 56], such as the surface plasmon resonance (SPR), surface enhanced Raman scattering (SERS), photothermal therapy, and even as a contrast enhancer in X-ray imaging; and thus, facilitating the recognition and sensing of biological molecules in complex samples compared to conventional methods [52, 53, 57–59]. Altogether, GNP have been demonstrated to have a multifunctional use as drug delivery, targeting, amplifiers, and even biomarkers being utilized in therapy, diagnoses, or the combination of both, the so-called theragnostic [49, 60]. Thus, GNP have become an interesting tool or complement to enhance conventional methods or techniques in order to increase their sensitivity, specificity, effectiveness, and safety. However, their use in preclinical studies and clinical trials for AD and PD is limited, and in the case of AD, they are mainly restricted to the Aβ peptide. Furthermore, the new therapeutic strategies for AD have shifted to include analyses that consider the Tau protein, and as with the α-syn protein, their relationship with GNP is scarce and has been poorly studied. Thus, both proteins have become an outstanding niche to develop research for new strategies and outlooks.

This review compiles and focuses on Tau and α-syn proteins as GNP targets, with a special attention to new functionalized nanosystems, how they can reach the central nervous system (CNS), and the detection at low concentrations of different Tau and α-syn species as biomarkers, including the current research in the oligomer field, to generate an early diagnosis. In addition, this review discusses not only in vitro and in vivo assays, state-of-art clinical studies, and FDA approvals, but also highlights the risks and concerns that underlie the uses and applications of GNP, as well as the latest advances and opportunities to generate and drive new methodologies to research the potential use of GNP with Tau and α-syn proteins for therapy, diagnosis, and theragnosis.

### Obtention of GNP

**Fig. 2:**
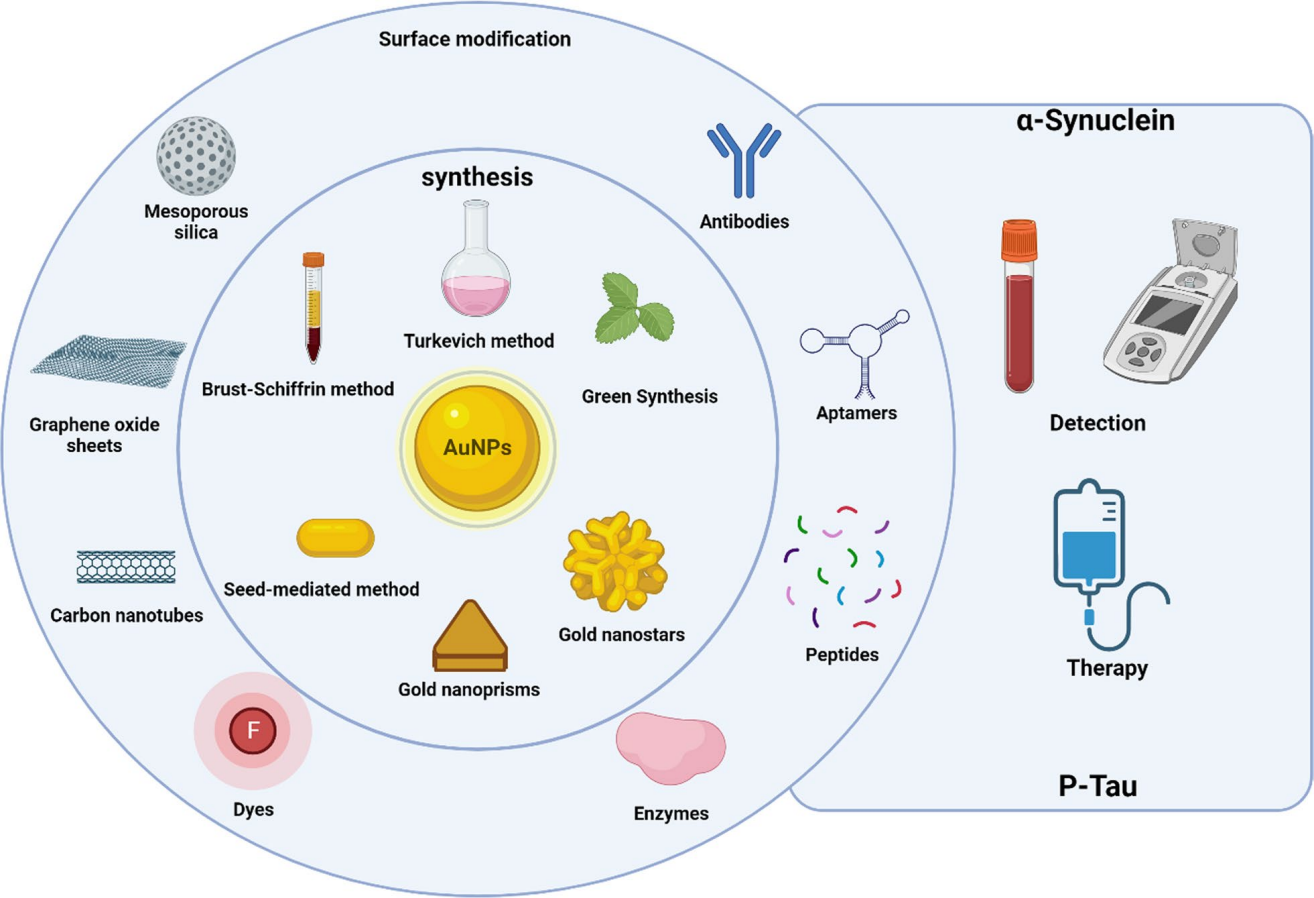
GNP can be obtained by several methods that are shown in the central circle (Turkevich, Brust-Schiffrin, and green synthesis specifically for GNS), obtaining different shapes and sizes that depend on the methodology used. These GNP can also be functionalized with various types of molecules to target α-syn and Tau proteins, making them interesting tools to generate novel strategies to apply in the detection and therapy of PD and AD, respectively. Created with BioRender.com

GNP have been widely used for electronic, optical, and biological applications including targeted drug delivery. GNP exhibit different geometries (Fig. 2), which can influence the interactions at the bio-nano interface, the functionalization degree, the molecular organization of these molecules as well as their chemical state, and even their biological uptake degree [61, 62]. Thus, the size and shape are fundamental for the chemical and surface properties, being a key part of the experimental design of a GNP [61, 63]. For these reasons, various methods have been developed to modulate these physical properties to obtain different geometries [64]. In this sense, Gold nanospheres (GNS) are one of the most studied shapes, possessing different synthesis methods such as Turkevich method [65], which involves citrate as a reducing and stabilizing agent to modulate their size [66–68]. Another strategy is the Brust-Schiffrin method, which allows to obtain small nanoparticles in the 1–3 nm range [69], but this synthesis generates a considerable amount of organic solvent used as a stabilizing agent.

Currently, the synthesis methods aim to develop a GNS synthesis more environmentally friendly and safe. Green synthesis involves the use of natural sources of chemicals to synthesize GNP [70, 71], avoiding the use of toxic chemicals and reducing waste products. These methods are mainly based on the presence of alkaloids and polyphenols, among other elements, from natural extracts, microorganisms, and molecules that allow the reduction of Au^3+^ to produce GNP. For example, polyphenols in the aqueous extract of *Abutilon Indicum* leaves [72] or alginate [73], a marine algae polysaccharide, have been used to synthesize GNS. However, these methods exhibit some limitations in polydispersity control because of the variety of molecules present in the extract or microorganism [74, 75]. To overcome these issues, the use of purified phytochemicals allows to obtain greater reproducibility, compared to the use of extracts, because there is an understating of which molecules participate as stabilizers and reducing agents [76]. Curcumin has been used to obtain GNS with promising effects in a human colorectal adenocarcinoma (HT29) cell line model [77], as well as their antioxidative and antimicrobial properties [78–80]. Gallic acid is another successful case used to synthesize GNP. This phytochemical is present in tea leaves, fruits, and nuts and has been used to synthesize GNS with a potential application as an antitumoral or anti-aging agent [81–83].

#### Anisotropic nanoparticles

Anisotropic nanoparticles are another area of research that has been developed because the shape drives their properties, and it also can be modulate with potential biomedical applications (Fig. 2). One of the most studied nanoparticles is gold nanorods (GNR) which can present their plasmon within the first and second biological windows, making them interesting for their applications in biomedicine [84–86]. The most common synthesis is the seed-mediated method using CTAB as a stabilizer agent [86], allowing to modulate the plasmon maximum, absorption, and scattering efficiency of the nanosystem [87, 88]. Gold nanoprisms (GNPr) also exhibit a plasmon that can be modulated in the biological window [89], but their synthesis is more friendly using sodium thiosulfate as a reducing and stabilizing agent [90]. Also, they scatter the light efficiently due to the greater number of hot spots, making them potential candidates as fluorescent probes in imaging [91]. Gold nanostars (GNSt) are another type of GNP that exhibit more hot spots than GNPr depending on the number of points and length of the arms presented, making them very interesting for their applications in plasmon-enhanced fluorescence (PEF) and surface-enhanced Raman spectroscopy (SERS) [91, 92]. Their synthesis is based on the use of HAuCl_4_ at basic pH and hydroxylamine as a stabilizer and reducing agent [93].

Hollow gold nanoparticles (HGNP) are another attractive type of nanoparticle because of their thickness and cavity size can be modified, allowing the maximum absorption to shift in different regions of the spectrum with potential application in photothermal therapy [94–96]. Also, their cavity can be used to encapsulate drugs, which makes it useful for drug delivery [97]. Their obtention is by templates over which Au is deposited from a HAuCl_4_ solution. Then, these templates are eliminated, and the size and shell thickness depend on the template used as well as the concentration of the added HAuCl_4_ solution [98]. Finally, even though anisotropic GNP exhibits great potential in many different areas, it is noteworthy that their obtention by green methods is still recent. Some issues related to their shape homogeneity and yield need to be improved and encouraged to develop an alternative to traditional chemical methods. For a complete revision see Table 1, and Ref. [99] and [100].

**Table 1:** Comparison of different synthesis to obtain GNP

Methodology	Toxic waste	Reproducibility	Scalability	Implementation
Templated	** + **	** + **	**−**	**−**
Electrochemical	**−**	** + **	**−**	**−**
Seed growth	**−**	**−**	** + **	** + **
Ecosynthesis	** + **	**−**	** + **	** + **
Physical Methods	** + **	** + **	**−**	**−**

Because the control of morphology, size, and distribution is not trivial [48], these physical features may modify the biological effect at the nano-bio interface, resulting in different behavior of the nanosystems in in vitro approaches compared to in vivo analysis. Nowadays, efforts are focused on developing smart nanosystems with optimized properties to increase the responsiveness of biological systems [61]. However, some concerns emerge for their use in biological systems related to their stability and how GNP may interact or behave with the medium that surrounds them in physiological conditions. Also, different synthesis uses organic compounds that are incompatible with their application in biological assays, affecting their potential use in diagnosis and therapy [51, 101–103]. To overcome this issue, the surface of GNP can be functionalized easily with different molecules, adding new properties, giving more stability, increasing their biocompatibility and half-life (e.g., plasma), and enhancing their selective delivery to organs.

In this sense, polyethylene glycol (PEG) is the most common way of eliminating toxic stabilizer agents or residues from the surface of GNP by a coating process [104]. Also, PEG increases the half-life of GNP in the bloodstream due to their hydrophilic nature, conferring water solubility, physiological stability, and biocompatibility to the GNP [105–107]. Another widely used option is a silica shell, which has been reported to enhance GNP stability and biocompatibility for biomedical applications [51, 108, 109]. Silica coating is chemically inert and allows the encapsulation of different kinds of molecules, such as drugs, dyes, and antibodies, among others, as well as an effective drug release[108]. Another interesting approach to masking the GNP is the use of endogenous elements present in plasma is. This strategy consists of capping the GNP with serum albumin (SA), which is the most abundant protein in plasma. As reported, these nanosystems exhibit longer circulation times, biocompatibility, and low toxicity, making their use widely accepted in the pharmaceutical industry [110–112]. Finally, it is noteworthy that green synthesis has been reported to enable the synthesis of GNP as well as their coating, increasing their biocompatibility. Curcumin and Apigenin are highlighted due to their potential use in biomedical applications. Whereas GNP functionalized with curcumin increase their half-life in the bloodstream with potential applications in neurodegenerative diseases[113, 114], GNP coated with Apigenin exhibit anti-inflammatory and anti-cancer properties in a photothermal treatment [115].

### GNP delivery to the CNS

**Fig. 3:**
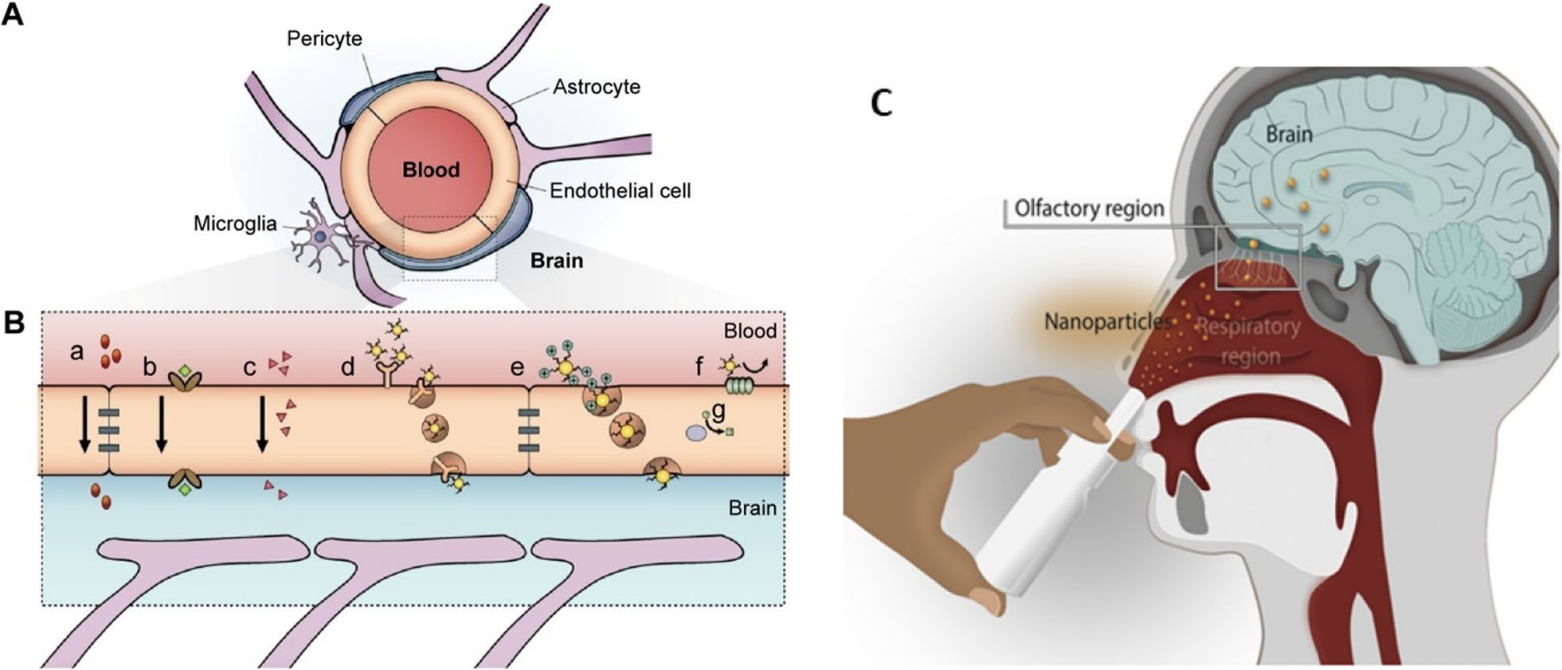
Principal pathways to overcome the BBB. **A** Scheme of the neurovascular unit of BBB constituted of pericytes, astrocytes, and endothelial cells. **B** The main mechanism of crossing through BBB: a) tight junctions restricting the pass of water-soluble compounds, b) carrier-mediated transport, [c] lipid-soluble agents, d) receptor-mediated endocytosis and transcytosis, e) adsorptive-mediated endocytosis and transcytosis, f) the efflux pump expulses the drugs from the endothelial cells to the blood, g) Cytochrome P450 enzymes. Adapted with permission from Ref. [126]. Copyright 2015, with permission from Dove Medical Press Limited. **C** Anatomical features of the intranasal route involved in nose-to-brain drug delivery. Adapted with permission from Ref. [133]. Copyright 2022, with permission from Elsevier

Currently, the engineering of GNP to deliver them comprises passive or active strategies for site-specific recognition [63]. The first strategy is based on nanoparticle accumulation into the brain depending on systemic circulation [47, 116, 117]. However, this mechanism is limited for the renal excretion, reticuloendothelial system, and immune system that eliminate the nanoparticles from bloodstream. Conversely, active targeting comprises the use of biological ligands functionalized onto GNP to recognize specific molecules on the cell surface (Fig. 3) [118, 119]. In fact, one of the current challenges is to assemble multiple ligands for delivery and targeting in one GNP. The best-case scenario is to design GNP in which the ligand merges both applications into one. Thus, the use of only one ligand is expected to yield a high selectivity for a particular body area, achieving better biocompatibility and ensuring the potential of the nanosystem for diagnosis and therapy.

Regarding neurodegenerative diseases, the engineering of nanoparticles must consider another factor to achieve an effective therapy, which is that GNP must be able to arrive in the CNS by crossing the BBB [120, 121]. This biological barrier controls brain homeostasis through a tight and sophisticated mechanism where layers of endothelial cells limit the entrance of different foreign molecules into the CNS (Fig. 3a). Thus, the BBB has become a formidable barrier that must be surmounted in the generation of treatment options for neurodegenerative diseases [122–124]. Several approaches have been designed, but most of them imply a disruption of BBB cells, making them invasive methods that result in deleterious consequences for the cells [123, 125]. For this reason, the use of GNP has emerged as a novel alternative because they exhibit different mechanism to cross through the BBB. For example, their internalization can be mediated by endocytosis involving both pinocytosis and phagocytosis mechanisms, but this mechanism results in a widespread organ distribution including kidneys and liver [126, 127]. To overcome this issue GNPs can be capped or functionalize with targeting molecules to delivery into the brain [128–130], being adsorptive-mediated transcytosis (AMT) and receptor-mediated transcytosis (RMT), being RMT the most specific (Fig. 3b). Peptides Angiopep-2 and CLPFFD-THR functionalized onto GNP are successful examples of RMT [131, 132]. The present section describes and summarizes the advances in the field of delivery and targeting, considering the use of GNP with Tau and α-syn proteins for AD and PD, respectively.

#### A new opportunity: the nose-to-brain delivery

Even though RMT is an interesting strategy, some drawbacks may reduce its effectiveness. Low bioavailability, inadequate permeation, delay in onset of action, and limited presence or saturation of receptors in the BBB are the most common issues. To overcome these limitations, intranasal administration (IN) has emerged as a non-invasive strategy to bypass the BBB, reaching and delivering drugs and nanoparticles directly into the brain (Fig. 3c) [133]. Via IN therapy the nanosystems enter by two main pathways: the olfactory and the trigeminal nerves [134, 135]. Due to the unique anatomical organization of the olfactory nerves, compounds can circumvent the BBB through two main mechanisms: 1) the neuronal endocytosis, in which compounds are internalized by olfactory neurons of the olfactory epithelium and transported through the axons to reach the olfactory bulb, and then other brain areas; 2) paracellular diffusion, in which compounds can diffuse through intercellular gaps in the olfactory epithelium and channels created between the ensheathing cells surrounding olfactory axons, being externally transported along the neuronal axon and reaching the CSF and olfactory bulb [134–136]. Regarding GNP, different studies have shown their effective delivery enhances treatment efficacy [137–140]. GNP can reach the olfactory bulbs in minutes [141]. Gallardo-Toledo et al. [140] analyzed GNS and GNPr functionalized with PEG and the peptide D1 (qshyrhispaqv), as a potential targeting for Aβ and their delivery into the brain. Also, it compared the IN with the intravenous route, demonstrating the major effectiveness of the IN route and reinforcing the idea that IN administration is a promising pathway for the delivery of GNPs into CNS. However, IN delivery has presented some concerns about mucociliary clearance that can easily eliminate the drugs administrated; therefore, some special formulations may be required to decrease this effect. To overcome this issue, GNP surface can be coated to specifically target the olfactory region, exhibiting mucoadhesive properties that allow a major retention and avoiding the mucociliary clearance. In this regard, chitosan emerges as an attractive mucoadhesive polymer due to its cationic nature, biocompatibility, and suitability for high loading of hydrophilic drugs [142]. For example, Bhumkar et al. loaded insulin in GNP coated with chitosan. After transmucosal administration, they were able to reduce glucose levels on the blood of rats, noting a higher increase in the insulin level after nasal administration compared to oral administration [143].

### GNP and diagnosis

**Fig. 4:**
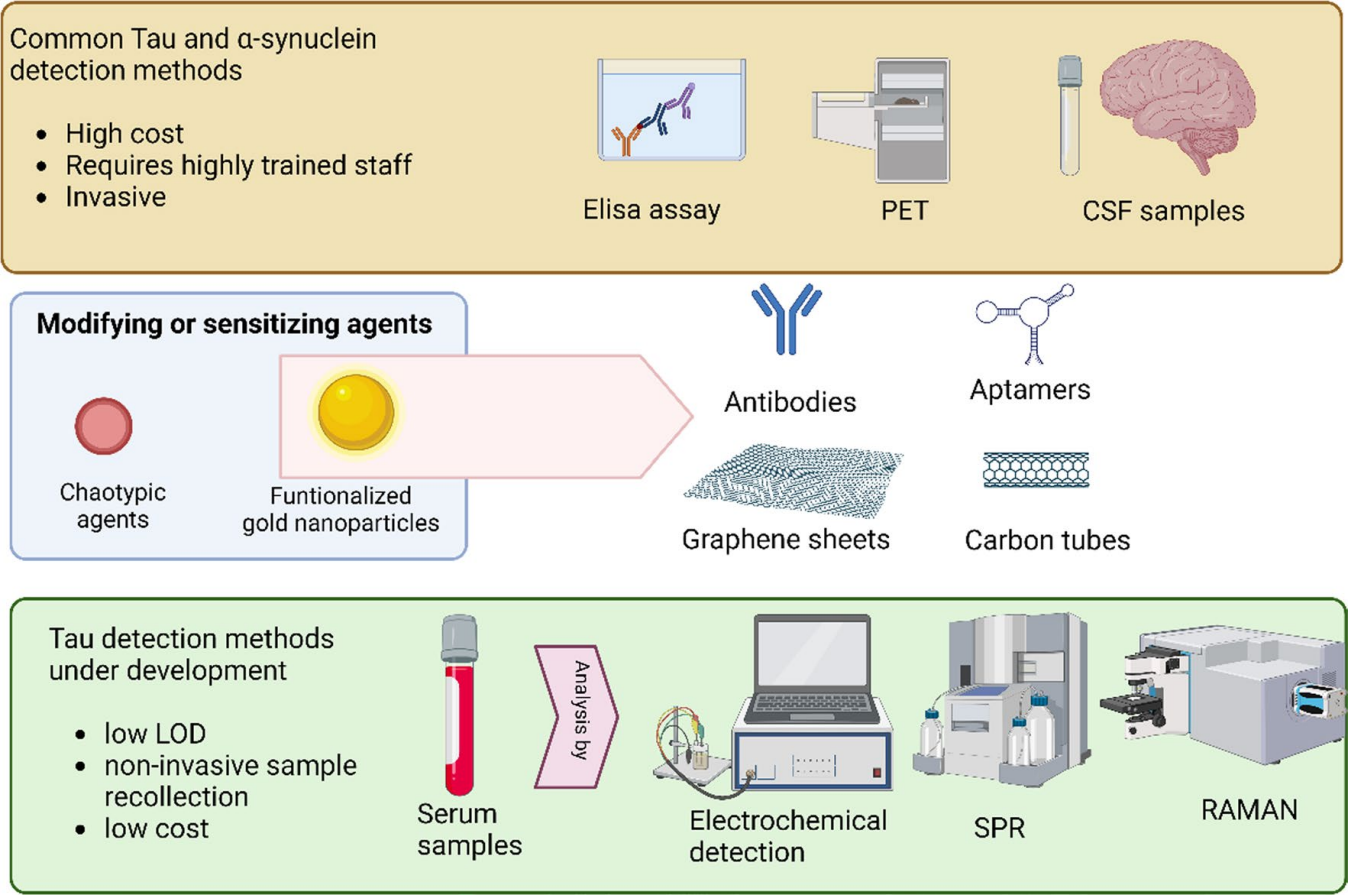
Conventional methods to detect Tau are often expensive, invasive, and require highly trained personnel. However, the use of nanoparticles is enabling the development of new detection methodologies that significantly improve the sensitivity and selectivity of the process. LOD: limit of detection. Created with BioRender.com

Tau protein is one of the main etiological agents of AD and other tauopathies [144]. Likewise, one of the key biofluid-based biomarkers for PD is the misfolded and aggregated α-syn protein [1, 145, 146], which is characteristic of Lewy bodies and hallmark of this disease [31, 147–149]. In this sense, both α-synuclein and Tau protein can be detected in CSF [144, 146], but this process involves the extraction of the sample by lumbar puncture, which is an invasive and painful procedure for the patient. Detection methods are currently limited to ELISA immunoassay and PET imaging (Fig. 4) [144]. The latter method is expensive, requiring access to a cyclotron, and uses radiotracers with a short half-life. In this context, research has been carried out to find new detection and diagnosis forms that are sensitive, economically accessible, and non-invasive [150–153]. Currently, researchers are investigating sensitive methodologies that allow the detection of Tau and α-syn in different biofluids. Numerous strategies based on GNP technology have emerged in the search for methodologies that allow the quantitative detection of both proteins (α-syn or Tau) with high sensitivity (for a complete review, see Table 2). Most of these biosensors are based on electrochemical methods, SERS, or localized surface plasmon resonance (LSPR) (Fig. 4) [154–156]. In general, these methods are based on immobilization on the surface of the GNP with a selective molecule for the target molecule (antibody, aptamer, etc.) and/or a reporter molecule. This strategy allows an improvement in both the limit of detection (LOD) and sensitivity (Fig. 4).

#### GNP designed for α-synuclein diagnosis

**Fig. 5:**
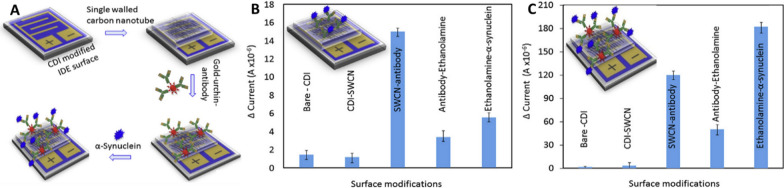
The presence of gold nanourchin increases the sensitivity of detection for α-syn. **A** Schematic representation for detecting α-syn on a single-walled carbon nanotube (SWCN)-modified IDE surface, assisted by goldnanourchin-immobilized antibodies. Detection of 1 nM α-syn using the platform in the absence (**B**) or presence of gold nanourchin (**C**). Adapted from Ref. [155]. Copyright 2020, with permission from Elsevier

In this regard, Zhang et al*.* [155] showed that modifying a single-walled carbon nanotube with a gold-nanourchin-conjugated α-syn antibody significantly enhanced the LOD of the biosensor. Specifically, the biosensor displayed a 1000-fold increase in sensitivity, detecting concentrations as low as 1 fM compared to the surface coated solely with antibody, which detected concentrations as low as 1 pM (Fig. 5). Another notable immunosensor for α-syn is based on dual signal amplification (enzyme-assisted signal amplification and electrochemical measurement). The sensor is constructed with PAMAM-Au nanocomposites and an immunosensor surface using a horseradish peroxidase-secondary antibody (HRP-Ab2) linked to GNP. This sensor has high analytical performance, sensitivity (LOD: 14.6 pg mL^−1^), and stability [156]. The approach of using nanocomposites is very interesting since it allows for anchoring larger amounts of target protein with high stability and bioactivity. Furthermore, these detection analyses must exhibit both high sensitivity and high specificity for clinical examination using complex samples such as human plasma and CSF. In this context, there are some reports, such as that of Aminabad et al*.* [157], who developed a system based on GNP-modified graphene for bioconjugation with a biotinylated antibody (bioreceptor). Like the previous one, this nanosystem exhibits a synergistic effect between graphene and GNP, resulting in enhanced electrochemical activity. Consequently, it enables the development of a high-sensitivity electrochemical immunosensor (LOD: 4 ng/mL) for human plasma. In addition, there is a very interesting study in which the CSF is measured using a neuro-biosensor system that consists of an electrode with GNP-PGA-modified ITO, with a LOD of 0.135 pg/mL [158]. Essentially, a concentration of α-syn at the picograms per milliliter (pg/mL) level has been reported in the CSF of healthy individuals; however, it increases to nanograms per milliliter (ng/mL) in PD patients [159]. Thus, unlike the previous biosensor that detected α-syn in human plasma, this neurobiosensor system can identify and discriminate α-syn in the CSF of both healthy individuals and PD patients with high sensitivity, making it a promising tool for the diagnosis of PD.

Early diagnosis is a key factor to evaluate in neurodegenerative diseases. Since the oligomer species are recognized as the most toxic species that appear in the first stages of the aggregation process, early detection of α-syn oligomer is necessary for PD or other neurodegenerative diseases [36, 160]. In recent research studies, aptamers have emerged in the search to identify the oligomers specifically. Aptamers are "artificial antibodies" selected from a randomized group of nucleic acids that are very specific and stable for the target molecule. For example, Wu et al*.* [161] successfully designed electrochemiluminescent systems based on GNP-cooper-metal–organic frameworks (NP-Cu-MOFs) with an aptamer as the recognition element for α-syn oligomer detection. In the search for an effective diagnosis of PD, the literature has described that a selective α-syn nitration is a modification associated with oxidative and nitrative damage of neurodegenerative synucleinopathies, and it has been found in biological fluids in patients with PD [162–164]. This has generated a new challenge for the design of diagnostic systems to detect the different conformations of α-syn. In this context, Zhang et al. [165] designed an electrochemical immunosensor as a sensitive sandwich assay that utilized supramolecule-mediated GNP composites (GNCs) with anti-α-syn-nitration magnetic nanoparticles (MNPs) as signal amplification tags (LOD: 310 pg mL^−1^). The significance of this work lies in the fact that it investigated the detection of α-syn nitration in diluted serum samples obtained from healthy donors and PD patients, indicating a significant difference between the two study groups and the potential use of this system for clinical applications.

#### GNP designed for Tau protein diagnosis

On the other hand, Tau protein is a key player in neurodegeneration that poses a diagnostic challenge. Some literature highlights such as Neely et al*.* [166], which synthesized GNP functionalized with an anti-Tau antibody (Tau-mab) that can detect up to 1 pg/ml of Tau protein, which is two orders of magnitude lower than the values found in CSF. A similar result was obtained using a monoclonal anti-Tau antibody-coated GNP based on a two-photon scattering assay, in which the sensitivity of the method increased about 16 times [166]. Kim et al. [167] investigated the use of guanidine chloride to enhance Tau detection in blood samples. In this case, the researchers demonstrated that the chaotropic agent guanidine chloride improved sensitivity and selectivity compared to traditional methods, preventing the interfering effects of other components in the biological fluid. This same effect was tested by LSPR using PEG-functionalized GNR and anti-Tau antibodies. Therefore, guanidine chloride allows differentiating samples from healthy individuals and patients with AD.

The development of sensitive, non-invasive diagnostic methods for neurodegenerative diseases, such as AD and PD, represents a crucial frontier in medical research. With advancements in systems based on GNP, it is possible to develop innovative approaches to detect biomarkers like Tau protein and α-syn with high sensitivity and specificity. Table 2 describes the main technologies currently developed as sensors to detect α-syn and Tau protein with potential diagnostic applications.

**Table 2:** Overview of developed sensors based on GNP for α-syn protein detection

Biomarker	Technique	Nanomaterial	Recognition	Linear range	Limit of detection (LOD)	Refs.
amyloid fibrils	Immunosensor photoelectrochemistry	Au-doped TiO_2_ nanotube	Antibody	50 pg mL^−1^ to 100 ng mL^−1^	34 pg mL^−1^	[154]
Monomers and amyloid fibrils	U-shaped fiber-optic LSPR	Chitosan-nanogold matrix	Chitosan	N.A	70 nM	[168]
α-syn fibrils	LSPR	GNR		N.A	1 nM	[169]
α-syn fibrils	Immunosensor voltammetry	Carbon-gold nanoururchin-modified nanotubes	Antibody	N.A	1 fM	[155]
α-syn oligomer in diluted serum samples	Electrochemiluminescence	GNP	Aptamer	2.43 fM to 0.486 pM	0.42 fM	[161]
α-syn	Interdigitated electrode	GNP	Aptamer	N.A	10 pM	[170]
α-syn oligomers	Photoelectrochemical (PEC)	GNP/graphdiyne and WSe2	Aptamer	10 aM to 1.0 nM	3.3 aM	[171]
α-syn	electrochemical immunosensor	PAMAM-GNPCs	Antibody	20 pg mL^−1^ to 200 ng mL^−1^	14.6 pg mL^−1^	[156]
α-syn in cerebrospinal fluid	electrochemical immunosensor	GNP- PGA	Antibody	4–2000 pg mL^−1^	0.135 pg mL^−1^	[158]
α-syn in human plasma	electrochemical immunosensor	GNP-Gr	Antibody	4 to 128 ng mL^−1^	4 ng mL^−1^	[157]
nitrated α-syn in blood	Ultra-sensitive electrochemical immunosensor	GNCs	Antibody modified mnps	1–1000 ng mL^−1^	310 pg mL^−1^	[165]
Tau-381 in human serum	photoelectrochemical sensor	GNP	Aptamer	0.5 fM - 1.0 nM	0.3 fM	[172]
Tau in human plasma and brain tissue extract	electrochemical immunosensor	3D-GNP-PAMAM	Antibody	N.A	1.7 pg/mL	[173]
Tau-441 in CSF	electrochemical impedance spectroscopy and cyclic voltammetry	rGO/GNP nanocomposite	Antibody	1–500 pg/mL	0.091 pg/mL	[174]
Tau-441 in CSF	Nano-iPCR	GNP	Antibody	N.A	5 pg/mL	[175]
Tau-441 Human plasma	SERS sensor	Gold nanopillars	Antibody	10 fM^−1^ µM	3.21 fM	[176]
Tau and Aβ(1–42) CSF	SERS sensor	DNA-GNP conjugates	aptamer	N.A	4.2 × 10^–4^ pM (Tau) 3.7 × 10 ^−2^ nM (Aβ)	[177]
Tau and TDP-43 Human plasma and brain tissue extract	Electrochemical sensor based on sandwich-type immunoassay	3D-Gold-PAMAM	Antibody	N.A	2.3 pg/mL (Tau) 12.8 pg/mL (TDP-43)	[178]
Tau, Aβ(1–40), Aβ(1–42)	Shape-code nanoplasmonic biosensor	GNR	Antibody	N.A	34.9 fM (Aβ(1–40) 26 fM (Aβ(1–42) 24.6 fM (Tau)	[179]
Tau (60 kD)	LSPR-based immunochip	GNP	Antibody	N.A	10 pg/mL	[180]
Tau381 Human serum	Glassy-carbon electrode/carboxyl	GNP	Aptamer	N.A	0.70 pM	[181]
Tau (65 kD)	SERS-based sandwich assay	Modified MNPs	Antibody	N.A	25 fM	[182]
Aβ and Tau Human blood samples	3D SERS platform	(1D) CNT, (2D) GO, and (0D) plasmonic nanoparticle	Antibody	N.A	500 fg/mL (Aβ) 0.15 ng/mL (Tau)	[183]
Tau	Two-photon scattering assay	GNP	Antibody	N.A	1 pg/mL	[166]
Tau	SERS-active immunosensor	FeXOy and GNP	antibody	N.A	25 fmol/L	[184]
Tau-381 Serum samples	Electrochemical aptamer-antibody sandwich assay	Cysteamine-stabilized GNP	Antibody aptamer	N.A	0.42 pM	[185]
Tau-441 Serum samples	Multi-amplified electrochemical biosensor	MWCNTS	antibody	N.A	0.46 fM	[186]
Human Tau and Aβ(1–42) oligomers	Cd/Se/CdS/ZnS QDs and GNR-PDA	GNR-PDA	aptamer	N.A	20 pM (Tau) 50pM (Aβ)	[187]
P-Tau (181; 181,396; 404)	Raman scattering dual-mode magnetic immunosensor	GNP	Antibody	N.A	1.5 pg/mL	[188]

*N.A* not available

### GNP designed for targeted therapies

The therapeutic potential of gold nanoparticles (GNP) in neurodegenerative diseases like Alzheimer's and Parkinson's is being explored. Researchers are leveraging the unique properties of GNPs to develop innovative approaches for more effective diagnosis and treatment, opening up new avenues for theragnosis.

#### Targeted therapies for α-synuclein

The accumulation of α-syn protein plays a central role in the pathogenesis of PD. Liu et al*.* [189] developed a nanosystem consisting of GNP-pDNA-Lipo-NGF-DHA to deliver plasmid DNA (pDNA) to inhibit α-syn expression. Liposomes (Lipo) are the carriers of the GNP-pDNA, and Nerve Growth Factor (NGF) and docosahexaenoic acid (DHA) are the targeting molecules in the nanosystem. NGF has an important role in the maintenance and growth of neurons, which have NGF receptors in their membranes, promoting the entrance of GNP into the neurons [190]. DHA is a polyunsaturated fatty acid that favors memory and cognitive functions, and it also promotes the movement of GNP across the BBB due to the presence of DHA receptors in the membrane of the BBB. This nanosystem shows effective GNP delivery into the CNS, reducing the overexpression of α-syn and improving motor dysfunction and exploration abilities in a PD mouse model. Hu et al. [191] developed a similar CTS@GNP-pDNA-NGF nanosystem. In this case, chitosan (CTS) works as a scaffold in which its cationic groups bind to pDNA, inhibiting of α-syn transcription and protecting from enzymatic digestion. This approach demonstrated effective delivery of GNPs to the central nervous system, significantly reducing α-syn overexpression and improving motor behavior in animal models of the disease.

**Fig. 6:**
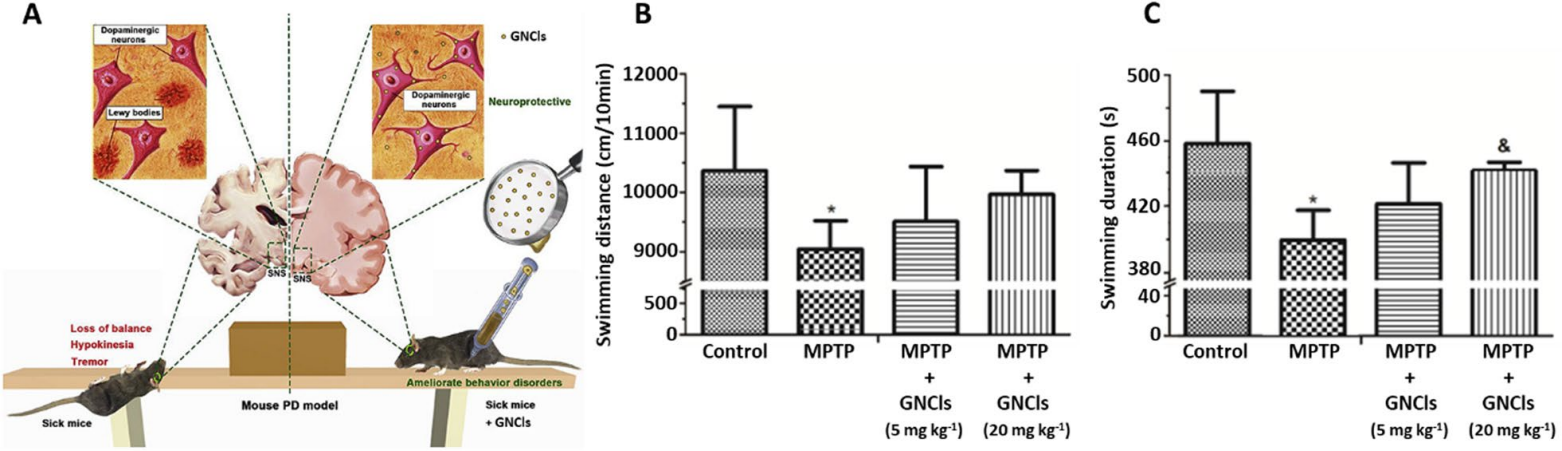
Treatment of GNP in in vivo transgenic mouse models. **A** Experimental design of treatment with GNCls in a PD model. Effect of GNCls on motor coordination of mice in a mouse PD model using a swimming test. **B** Evaluation of swimming distance. **C** Evaluation of swimming duration. Adapted with permission from Ref. [192]. Copyright 2019, with permission from John Elsevier

Additionally, Gao et al*.* [192] synthesized gold nanoclusters (GNCls) functionalized with N-isobutyryl-L-cysteine (L-NIBC) for the prevention of α-syn fibrillation in vitro. Furthermore, in vivo experiments using mouse PD models showed amelioration of behavior disorders with this nanosystem. The anti-PD effect of the L-NIBC-GNCLs system was evaluated using MPP + lesioned cells, and the in vivo models consisted of MPTP-induced mice, being MPTP a molecule commonly used to induce PD in animal models. The cell studies showed no obvious toxicity and a significant decrease in apoptosis compared to the MPP + control, demonstrating the neuroprotective effect of the GNCls. For the in vivo studies, the MPTP-induced mouse PD models were treated with different doses of the nanosystem administered via intraperitoneal injection. The results showed an improvement in locomotor activity in the mice in the presence of GNCls, significantly increasing the speed and distance traveled, thus, proving the effectiveness of the nanosystem and its delivery (Fig. 6).

#### Targeted therapies for Tau Protein

Numerous therapeutic studies have focused on Tau protein, which is pivotal in neurodegeneration. Due to the limited studies conducted with GNP, we have included in the review studies involving other nanoparticles and approaches, which may serve as a guide for developing theragnostic using GNP.

**Fig. 7:**
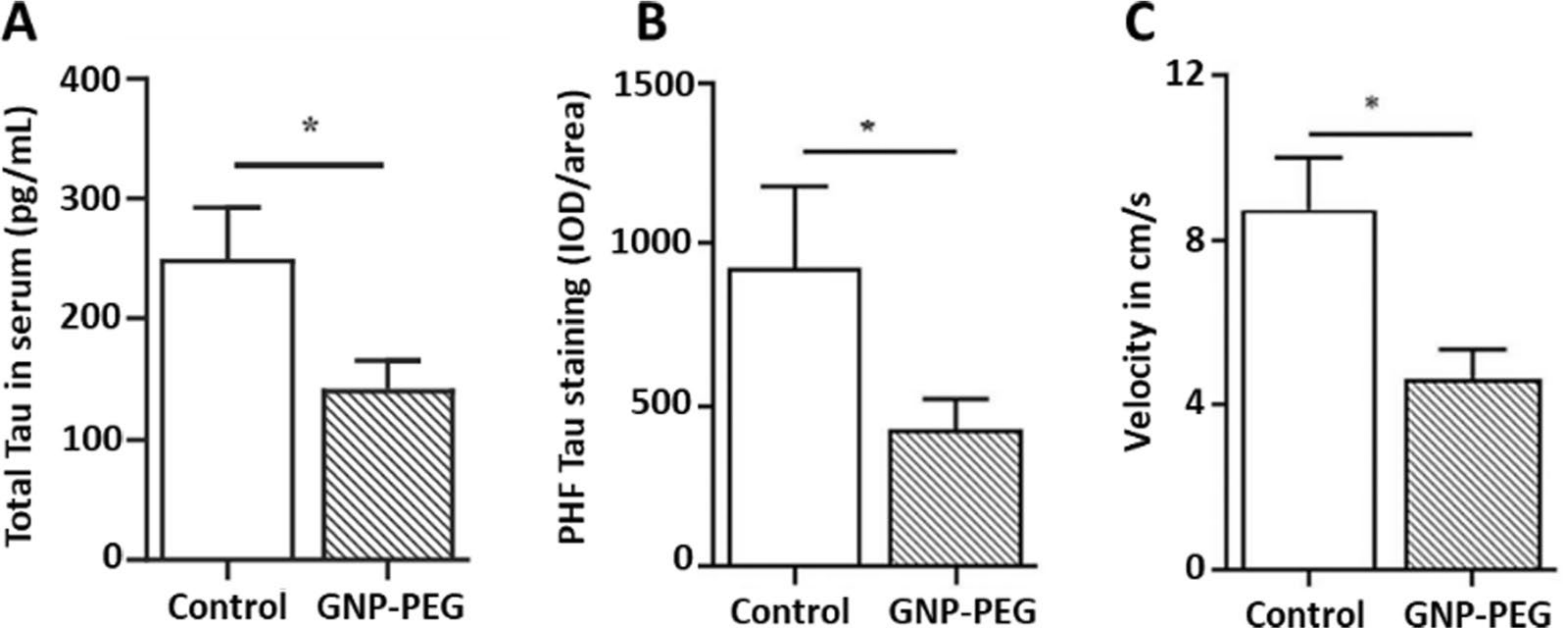
**A** Quantification of total Tau concentration in serum samples of control and GNP-PEG-treated tau P301L mice using an ELISA assay after 30 days of treatment. **B** Quantification of integrated optical densities (IOD) values in control (n = 6) and treated groups (n = 6) for paired helical filament-positive cells using Image-Pro Plus analysis. **C** Novel object recognition test for the examination of learning and memory, in which the mice exhibited altered velocity. Adapted with permission from Ref. [194]. Copyright 2020, with permission from John Wiley and Sons

In this sense, Sonawane et al*.* developed two interesting types of protein-capped (PC) metal nanoparticles (PC-F_3_O_4_ and PC-CdS) for the inhibition of Tau aggregates, both obtained via biological synthesis with two fungal species (*Funsarium oxysporum* and *Verticillium* sp), showing that PC-CdS inhibited Tau aggregates by 63% and PC-F_3_O_4_ by 49% [193]. Gao et al*.* loaded Curcumin onto red blood cell membrane-coated PLGA nanoparticles bearing T807 molecules as an imaging agent for positron emission tomography. This nanosystem was able to cross the BBB, and curcumin reduced P-Tau levels and neuronal death both in vitro and in vivo. Vimal et al. [194] investigated the therapeutic potential of GNP functionalized with PEG in transgenic Tau P301L mutant mice and macaque monkey serum (Fig. 7). These nanostructures acted as pseudo-nanochaperones interfering with Tau aggregation and significantly improving neuronal health in transgenic models. Regarding the ex vivo experiment using monkey serum the authors observed that this nanosystem interfering with Tau aggregation. Additionally, Bhattacharyya et al. posited that GNP could act as nanochaperones [195]. GNP capped with citrate of approximately 5 nm were suggested to allow remodeling of the P301L mutant Tau protein in vitro, modifying its structure and recovering the transport function of the microtubules. Thus, these studies highlight the role of GNP in modulating Tau pathology and maintaining brain function.

#### Opportunities in therapy

Even though the studies related to the targeting and delivery of Tau protein are scarce, some strategies may be applied or replicated from the research using the amyloid cascade theory, and GNP functionalized with different molecules. Liu et al*.* [6] functionalized GNR with the APH (ST0779) enzyme and the scFv (12B4) antibody, which binds to Aβ oligomers and fibrils and can cross the BBB. Javed et al*.* [196] also used GNP coated with β-casein, which binds to Aβ and can cross the BBB in zebrafish larvae and adult models via intracardial and cerebrovascular administration. In this sense, Martins et al*.* [197] functionalized GNP with Apolipoprotein E3 (ApoE3), which promotes both selective interaction with Aβ aggregates and can also cross the BBB. Altogether, if these approaches are used in a Tau protein context, they allow reaching the CNS.

Another potential strategy to replicate is the use of GNP functionalized with specific D-enantiomeric peptides, which are produced by mirror-image phage display to detect specific targets [198, 199]. These peptides exhibit several advantages for their use in biological systems, such as that they are not recognized by proteases, making them more resistant to degradation and increasing their half-life [200]. Interestingly, these D-peptides can cross the BBB and decrease both the amyloid load and neuroinflammation in transgenic models of AD [201–208]. Various studies have enhanced the inhibitory effect of these peptides on Aβ using GNP across multiple models (in vitro, in vivo, and even ex vivo) [84, 85, 209]. This progress highlights efforts to develop GNP-based systems for delivering targeted molecules, including these peptides, through the BBB for potential noninvasive CNS treatments. In this way, the use of enantiomers emerges as an interesting strategy to use with Tau protein. Recent reports have indicated that D-peptides can target Tau protein with a high inhibitory effect on the Tau aggregation process, and show valuable specificity, low toxicity, and high penetrance into neuron cell cultures [210–214], as was observed, for example, with the d-ttslqmrlyypp sequence [213]. Similarly, some studies have explored the use of D-peptides to disrupt the α-syn aggregation process. Shaltiel-Karyo et al. [215] found that β-syn exerts an inhibitory effect on α-syn. They designed a retro-inverso analogue of β-syn with high stability in mouse serum, which inhibited α-syn aggregation in in vitro and in vivo assays. Chemerovski-Glikman et al. [216] developed a self-assembled cyclic D,L-α-peptide which inhibited α-syn aggregation and even reduced its toxicity and intracellular accumulation. Horsley et al. [217] compared the effects of a D-peptide and an L-peptide to prevent the α-syn aggregation process, demonstrating that a D-peptide (d-gvlyvgs) was more effective in leading the α-syn monomers into a less aggregation-prone state, alleviating the cytotoxic effects of α-syn aggregates in cell models. Furthermore, these peptides have also exhibited the ability to cross the BBB and high stability in biological fluids, making them a promising strategy to develop new therapeutic agents. Thus, D-peptides open an interesting niche to functionalize them onto GNP as an effective future therapy. Thus, the studies with Aβ peptides can serve as models to follow for generating new treatments, considering the already described benefits as a guide.

Interestingly, since Tau protein is known for its close relationship with the microtubule network, it has also been the subject of interest as a target for treating diseases such as cancer. Ghalandari et al. [218] proposed the use of GNP or Fe_3_O_4_ nanoparticles coated with gold and functionalized with folic acid for photothermal treatment. The results of this study indicated that GNP interact with Tau and tubulin, preventing the formation of long polymers, and triggering apoptosis of malignant cells. Goto et al. [219] functionalized GNP with anti-Tau and anti-histone H1 antibodies to obtain high-resolution images that allowed the study of intracellular elements for protein localization [174]. Finally, Chia-Hsiung et al. [220] presented evidence that Plasmon-Activated Water (PAW) reduces β-amyloid and P-Tau burden in murine models of AD (APP/PS1), improving the behavioral parameters. In this article, the authors used resonantly illuminated GNP, reducing the hydrogen bonds present in the native structure of water and allowing PAW to be obtained. PAW could trap free radicals, which is one of the main mechanisms through which the damage produced in AD would be reduced in the model used. These results emerge as interesting approaches for a field that has not been sufficiently explored, along with their applications in neurodegenerative diseases, allowing the development of potential therapies.

### Current clinical trials, GNP, and FDA

Nanomedicine research has expanded greatly in recent years, resulting in potential medical and pharmaceutical tools and systems that are either FDA-approved or in clinical trials. There are currently 537 clinical studies registered on Home-ClinicalTrials.gov referring to nanoparticles, including polymeric, metallic, and lipid nanoparticles, among others. Of these clinical studies, only 7 are clinical trials aimed to neurodegenerative diseases: 4 trials of GNP, 2 of lipid nanoparticles, and 1 of polymeric nanoparticles (Table 3). This data shows the huge difference between GNP and other types of nanoparticles, and the inconsistency between the preclinical and clinical studies. There is a large literature related to preclinical assays of GNP, but their clinical translation is scarce [221, 222]. In this sense, some concerns support this evidence. One of the reasons is that most research in this field is related to material science, where the main objective is to generate new materials with new properties instead of exploiting the already known materials. For example, the synthesis of GNP is commonly simple, easily scaled up, and tunable, and GNP can be functionalized with different kinds of molecules, drugs, etc., as was above mentioned. However, despite these promising features, clinical studies with these nanostructures are not common. One successful example is the AuroLase Therapy, which was the first FDA-approved inorganic material. AuroLase Therapy is based on gold nanoshells which accumulate preferentially in cancerous tissue and using a near-infrared laser the affected area is irradiated, destroying the cancer cells by photothermal therapy [223]. Contrarily, among the clinical studies conducted with GNP, only one focused on PD while there are no records of GNP-based treatments for AD (Table 3). Although this study is a great advance for nanomedicine in its clinical applications, the use of nanosystems based on GNP for the detection of α-syn or P-Tau in clinical settings has not been reported in spite of the fact that these biomarkers are relevant for PD and AD, respectively.

**Table 3:** Current clinical trials addressed with nanoparticles for the treatment of neurodegenerative diseases

Name	NP type	Application	Clinical trial
APH-1105	Lipid nanoparticles	Alzheimer Disease	NCT03806478; Phase 2; Not yet recruiting
CNM-Au8	Gold nanocrystals	Amyotrophic Lateral Sclerosis	NCT04098406; Phase 2; Completed
CNM-Au8	Gold nanocrystals	Parkinson's Disease	NCT03815916; Phase 2; Completed
CNM-Au8	Gold nanocrystals	Amyotrophic Lateral Sclerosis	NCT04081714; Available
NTLA-2001	Lipid nanoparticles	Transthyretin-Related (ATTR) Familial Amyloid Polyneuropathy Transthyretin-Related (ATTR) Familial Amyloid Cardiomyopathy Wild-Type Transthyretin Cardiac Amyloidosis	NCT0460105; Active; Phase;1 not recruiting
CNM-Au8	Gold nanocrystals	Amyotrophic Lateral Sclerosis	NCT03843710; Phase 2; Withdrawn
Chitosan Phonophoresis	Chitosan nanoparticles gel	Device: Chitosan Phonophoresis Device: Therapeutic Ultrasound Device: Splinting Other: Neural mobilization exercises of the ulnar nerve	NCT05212311; Not Applicable; Completed

Another reason points to some concerns related to GNP toxicity and long-term accumulation [224]. However, GNP have been functionalized with different molecules to decrease their interaction with plasma proteins, increasing their targeting, and thus, showing a reduction in their side effects. For example, one clinical trial analyzed GNP functionalized with a siRNA for gliobastoma [225, 226]. This GNP-based therapy exhibited interesting results in patients treated for six months with tolerable side effects (which disappeared at the end of the treatment) being capable of crossing the BBB, promoting tumoral cell apoptosis, and tumor shrinkage. This example shows the potential use of GNP, their specificity, and in which their benefits outweigh the side effects.

**Fig. 8:**
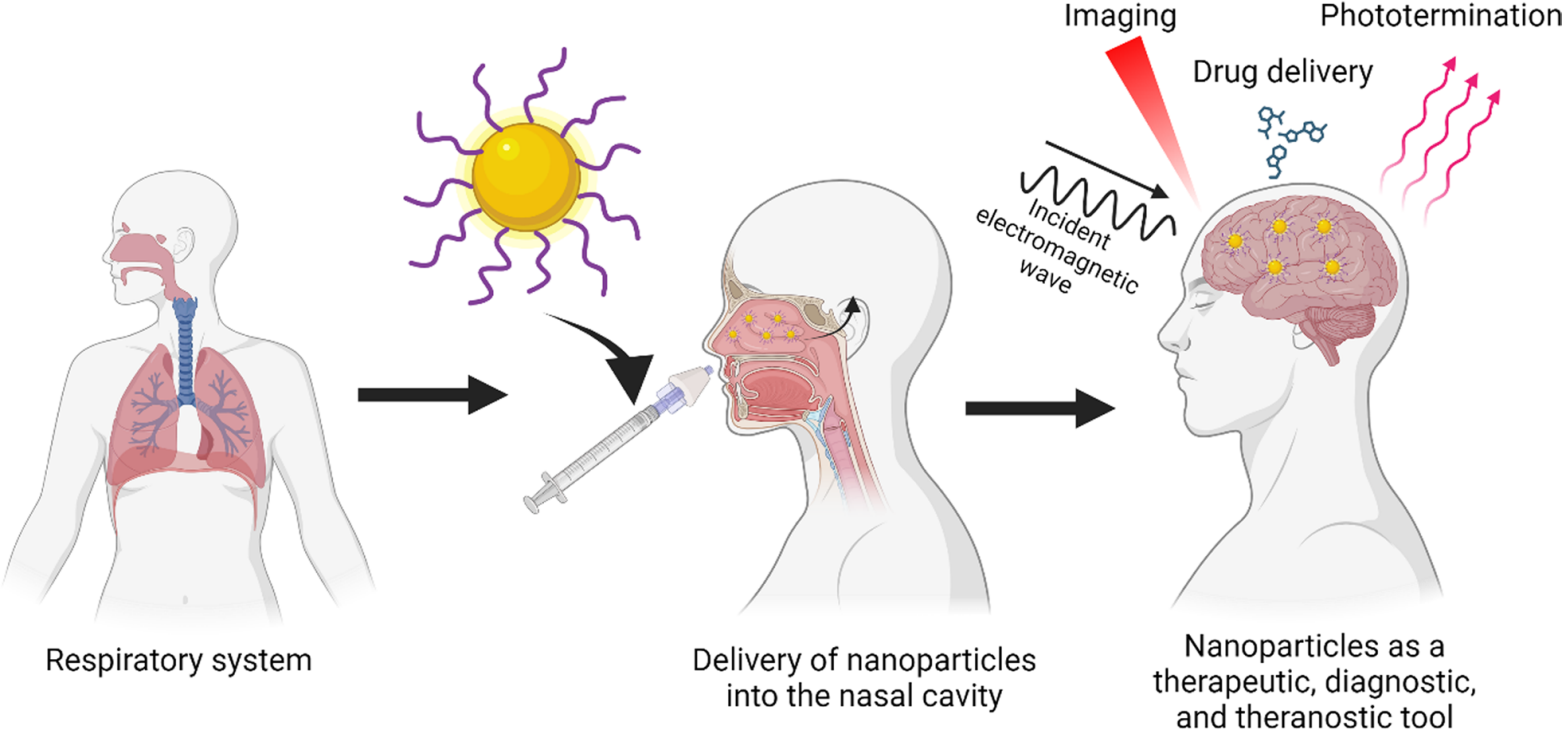
Overview of the ideal type of GNP administration for different applications such as therapy, diagnosis, and theragnosis to reach the CNS for the treatment of neurodegenerative diseases. Created with BioRender.com

In general, these sections collected and highlighted key literature contributing to the engineering of GNP and the development of a critical rational design to generate adequate analysis for targeting, delivery, diagnosis, therapy, or theragnosis. However, there is still another relevant point to address: the cytotoxic effect in elderly patients [227, 228]. It is known that their brain homeostatic capacity is reduced, and there is a lack of studies related to this age, requiring a further in-depth exploration of the toxic effect on this people. Thus, it is possible to generate a complete understanding of the GNP function in a physiological context. Strategies such as Nose-to-brain administration or the use of biodegradable nanosystems, such as those synthesized with poly-D-L-lactide-co-glycolide, polylactic acid, poly-e-caprolactone, or even natural materials such as chitosan [229] may help to minimize the possible toxic effects associated with bioaccumulation (Fig. 8) [140, 209, 230], improving their safety. Unfortunately, this research is still incipient and far from being analyzed in clinical trials. However, GNP are highly modifiable structures which their applications keep as potential and promising niches to explore and exploit to develop new therapies. In this sense, due to their tunable GNP nature, research that focuses on GNP behavior and their biological interactions can help to unravel and predict their potential effects and, therefore, raise the knowledge necessary for the rational design of gold nanosystems.

### Impact of GNP on the aggregation process: relevant issues for a rational design

Interestingly, another function of GNP is acting as chaperones due to their modulation, inhibition, or promotion properties exhibited in the amyloid aggregation process [231]. As explained above, the aggregation process can produce several species, such as oligomers, which are considered the most toxic species. Targeting the aggregation process using GNP becomes an interesting and potential strategy, not only because there are few reports in the literature regarding α-syn or Tau protein, but also because it is a potential niche to develop different therapeutic strategies for rational design. This has been widely observed and demonstrated using other kinds of amyloid proteins, such as amyloid-β [232–234] or β-microglobulin [235–237]. Thus, understanding the molecular mechanisms of the GNP-protein interaction may guide to develop new and alternative strategy to gain control over the aggregation process.

**Fig. 9:**
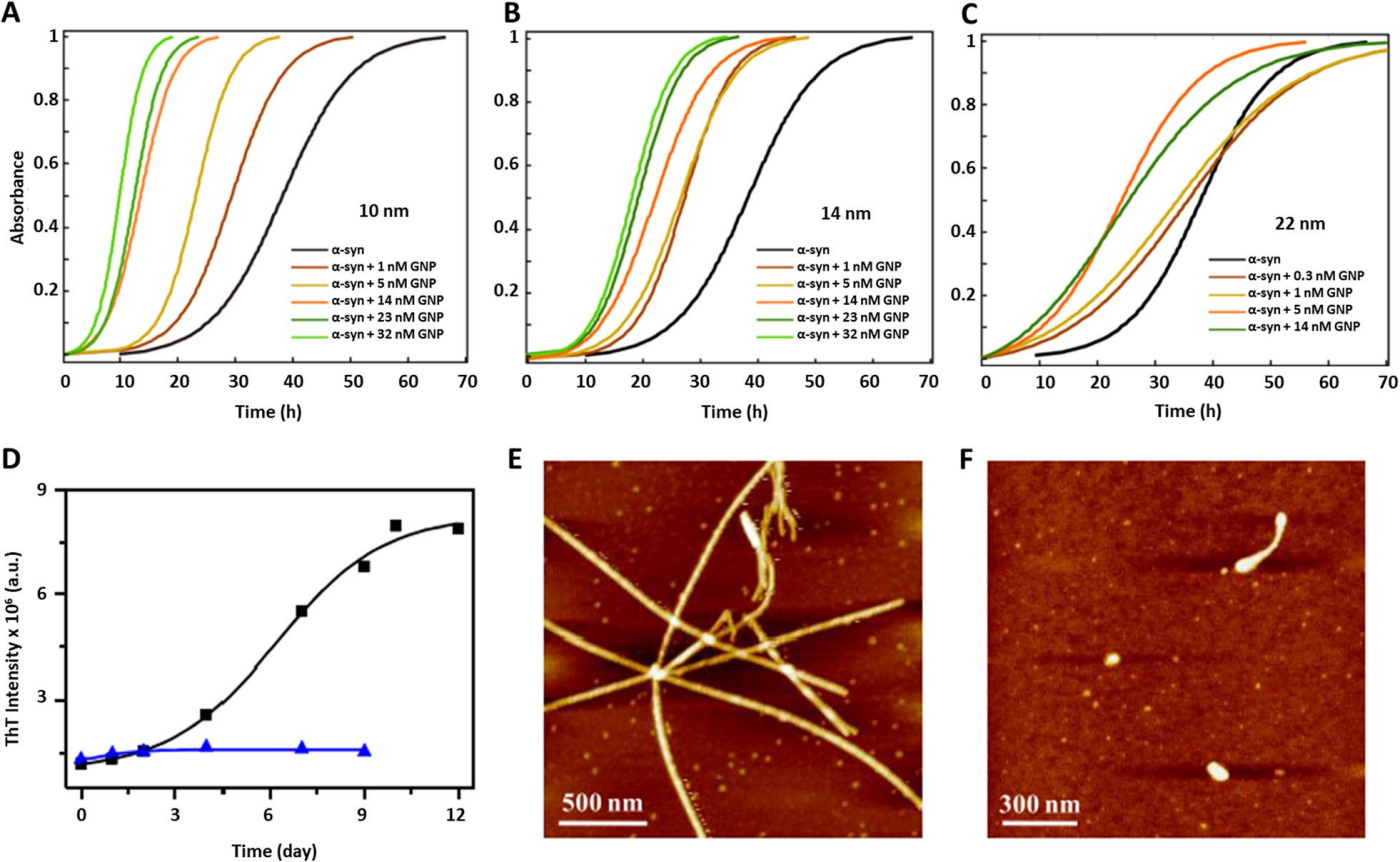
Kinetic assays of α-syn.** A**–**C** Kinetics of α-syn in the presence of different sizes and concentrations of GNS. Adapted with permission from Ref. [241]. Copyright 2013, American Chemical Society.** D** Kinetics of α-syn in the absence (black trace) and in the presence of GNS functionalized with porphyrin (blue trace). AFM images of α-syn incubated alone** E** and in presence of GNS functionalized with porphyrin** F** after 7 days. Adapted with permission from Ref. [242]. Copyright 2020, American Chemical Society

In this regard, one strategy widely used is monitoring the fibrillation process with ThT [238–240]. In Álvarez et al*.* [241], the authors analyzed the effect of different diameters and concentrations of GNS on the kinetics of α-syn, observing all sizes accelerated α-syn kinetics, but only 22 nm GNS were found to be preferentially bound to the fibril surface (Fig. 9a–c). On the other hand, in presence of GNS functionalized with porphyrin, there was not formation of fibrils, monitored by ThT and AFM [242]. Indeed, when this sample was analyzed by circular dichroism, α-syn remained in its normal α-helical pattern without the characteristic β-sheet signal, making it a potential therapeutic strategy (Fig. 9d–f). Another recent study was carried out by Maity et al. [243] using GNS functionalized with naringenin, a polyphenolic compound obtained mainly from several citrus fruits, showing an inhibitory effect on α-syn kinetics and stabilizing α-syn in its helix-α structure [219]. Similar results were observed using GNS functionalized with β-boswellic acid, a plant-derived terpenoid, which inhibit the Tau aggregation process [244].

Another type of GNP analyzed by kinetics assays is GNCls. This GNP have diameters of 2 nm or less, allowing them an efficient urinary excretion compared to larger GNP diminishing their secondary problems [245]. GNCls also exhibit a strong fluorescence in the visible and near-infrared spectrum and therefore, they have emerged as interesting tools to use in fluorescent bioimaging [57, 246]. GNCls functionalized with N-isobutyryl-L-cysteine exhibited an inhibitory effect on the aggregation process of α-syn studied by ThT [192]. Mahapatra et al*.* [247] also studied the capping-charge effect by introducing either a negative charge or an apolar agent on the surface of GNCls. Although both nanosystems decreased the neurotoxic effect of α-syn aggregates, they worked in different ways. Whereas negative GNCls inhibited the aggregation process, producing nonfibrillar aggregates, apolar GNCls accelerated the aggregation process, producing ribbon-like aggregates. Thus, both classes of GNCls guided the toxic aggregates towards less toxic species. Finally, the authors only analyzed the apolar GNCls in in vivo studies, which exhibited an increase in brain delivery and blood–brain barrier (BBB) permeability in a mouse model.

However, although GNP exhibit interesting properties and uses, they have shown different roles in peptide aggregation that could lead to deleterious biological consequences [248]. Specifically, GNP-protein interactions can alter how proteins behave on the GNP surface, modifying the protein adsorption and orientation and modulating their activity. [249]. Thus, their potential use requires a detailed analysis to clarify the impact of GNP on biological systems for generating a rational design of GNP. Yang et al*.* [250] studied the effect of negatively and positively charged GNP on α-syn orientation. They demonstrated that protein orientation depends on the surface charge: negatively charged citrate-coated GNP interacted with the N-terminal whereas positively charged poly(allylamine hydrochloride)-coated GNP (PAH-GNP) interacted with the C-terminal. These results were accompanied by an increase in the β-sheet content, suggesting that PAH-GNP induce conformational changes resembling the aggregation state of α-syn. A similar result was observed by Lin et al*.* [251]. GNP coated with (16-mercaptohexadecyl) trimethylammonium bromide (MTAB), a cationic agent, also promoted N-terminus exposure similar to PAH. However, α-syn interacts with MTAB-coated or PAH-coated GNP in different ways because of their respective intermolecular interactions. While MTAB is unable to create hydrogen bonds with α-syn, PAH contains ammonium ions that facilitate the formation of hydrogen-bonding interactions.

**Fig. 10:**
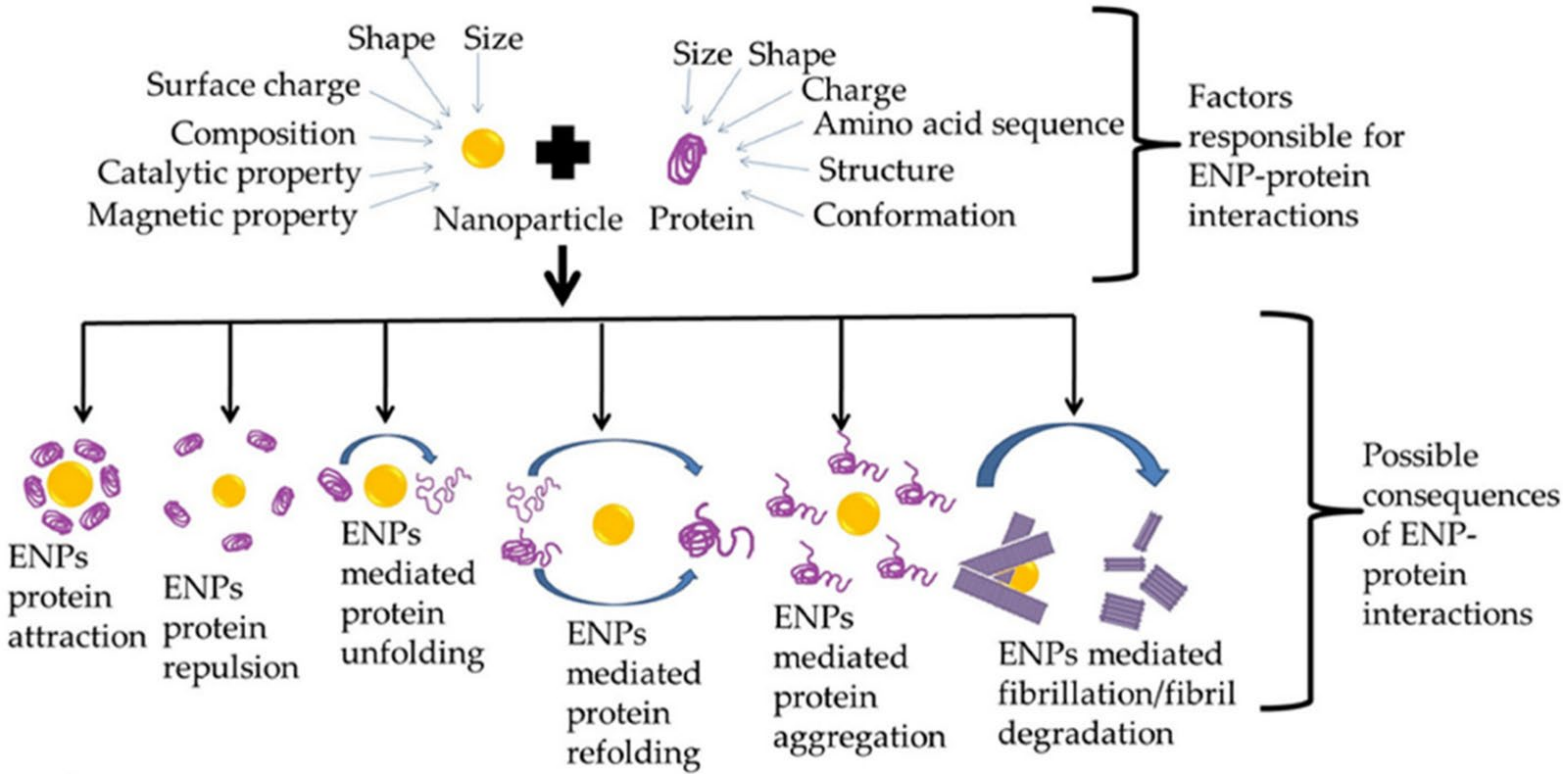
Different functions or consequences of engineering nanoparticles (ENP) interacting with proteins. As a product of this interaction, nanoparticles are capable of unfolding, refolding, promoting protein aggregation, or mediating the fibrillation or defibrillation process. Adapted with permission from Ref. [231]. 2022, by the authors. Licensee MDPI, Basel, Switzerland. This article is an open access article distributed under the terms and conditions of the Creative Commons Attribution (CC BY) license (https://creativecommons.org/licenses/by/4.0/)

Interestingly, McClain et al*.* [32] synthetized GNP coated with sodium dodecyl sulfate (SDS-GNP) to mimic the effect of biological membranes on α-syn conformation. This capping agent presents a negative charge that interacted with the N-terminus of α-syn, resembling the interaction of GNP with citrate. In fact, SDS-GNP were also found to expose the NAC region in a comparable manner to PAH-GNP. These results suggest that the effect of α-syn/GNP interaction could induce a higher α-syn propensity to aggregate depending on surface chemistry, modifying the orientation and adsorption of α-syn onto GNP. Therefore, determining this information becomes mandatory in order to predict and understand GNP behavior and their effects on biological systems and to apply this knowledge to develop future nanomaterials (Fig. 10).

### Challenges and potential risks of GNP use: neurotoxicity

The explosive development of GNP has raised interesting concerns related to their uses and applications. The surface and geometric design of GNP can modify or alter the biological responses both in in vitro and in vivo studies and in fact, the use of GNP on biological models is not free of limitations, risky factors, or side effects [252, 253]. In this regard, current research has been conducted on cytotoxicity analysis, and especially related to neurotoxicity [103, 254]. These studies have become relevant because any issue as a result of the use of GNP in the CNS has a major impact due to the low rate of regeneration of the CNS, and considering that the brain is a sensitive organ to the oxidative stress generated by GNP [130, 255]. Thus, the neurotoxicity of GNP has become a key parameter to improve and optimize nanostructures in order to design safer and non-toxic GNP for potential therapeutic applications in neurodegenerative diseases [256].

In fact, GNP have exhibited significant cytotoxic effects, such as generation of oxidative stress, disruption of the lipid bilayer, necrosis/apoptosis, mitochondrial dysfunction, and DNA damage [103]. These harmful effects are controversial because there is a wide spectrum of studies and analyses using different sizes and shapes of GNP, and there are also differences in the physicochemical nature of GNP coating and the doses or administration routes of GNP [103, 130, 253]. Thus, this wide variety of studies makes it difficult to generalize and point out the effect or behavior of GNP on biological systems, rendering this analysis nontrivial. In this sense, there have been numerous in vitro studies published in the literature describing the neurotoxic effects of GNP, but only are few in vivo studies have been reported [103]. Because the use and application of GNP have sparked an explosive growing interest in biomedicine, especially in CSN disorders, it is mandatory to disclose the major spectrum of neurotoxic effects that GNP can exhibit. Therefore, this review will concentrate on considering and collecting in vivo*, *in vitro*,* and also ex vivo studies that have documented the neurotoxic effects of GNP in order to display the current state-of-art research related to this key parameter.

Nowadays, the most common and widely studied GNP shape is the sphere. Different sizes, treatments, administration routes, coatings, and also the type of animal, their sex, and their age, are all parameters that make it difficult to assemble the results in order to surmise the effect of a particular GNP. Bare GNS with different sizes have been analyzed in in vivo studies, yielding somewhat conflicting findings. GNS with a diameter of 10 or 30 nm have shown neurotoxic effects [257], while GNS with a diameter of 12.5 or 50 nm have been reported to be non-neurotoxic [258]. Both studies were carried out with different GNP concentrations, types of animals, and treatments. Similar neurotoxic effects, learning impairment, biochemical changes, and a slight apoptosis and inflammatory response were exhibited in the case of GNS [259–261] administered either intraperitoneally or by a unilateral stereotactic injection into the cerebral cortex [262, 263]. Of note GNS with a diameter of 50 nm injected intravenously showed a non-neurotoxic effect [264]. Another kind of analysis reported is the ex vivo study using the patch-clamp method on CA1 and CA3 neurons. In this case, stars and spheres were analyzed, but both types of GNP provoked disturbances in neuronal functions similar to those found under pathological conditions [265, 266].

Interestingly, GNP can diminish these toxic effects with an appropriate coating [130, 252]. Although GNS and GNCls with different coatings have commonly exhibited a non-neurotoxic effect [267–269], some coatings have exhibited a slight decrease in cell viability in in vitro assays [270]. Regarding in vivo assays, GNS functionalized with imidazole exhibited a neurotoxic effect, whereas bare GNS did not impact cell viability [271]. Another example of the apparent contradictory effect of GNS is coating with PEG, which is the most common molecule used to increase GNP stability [272]. GNS-PEG showed a toxic effect in mice that was reverted by a second functionalization with Trolox, an antioxidant molecule [273]. However, GNS-PEG exhibited a non-neurotoxic effect compared to the bare GNS in rats [274]. This non-neurotoxic effect was also observed using different shapes of GNP functionalized with PEG in ex vivo assays [275, 276]. Another interesting analysis was carried out using GNS or GNCls with different coatings in a PD-induced model. These studies demonstrated that GNP exhibited a neuroprotective effect, reversing the deleterious damage observed in this disease [191, 192, 277], and showed an anti-neuroinflammatory effect compared to the controls [277]. This neuroprotective effect was also observed in a transgenic Tau mouse model of AD, in which the presence of GNP improved the cognitive functions of the mice (Fig. 12) [194].

**Fig. 11:**
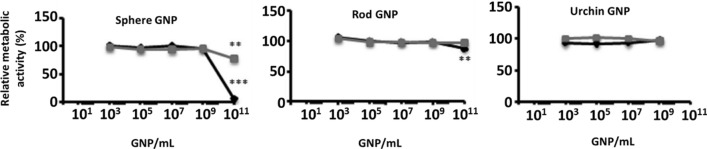
Cytotoxicity effect of GNP coated with either CTAB or PEG in a microglial (N9) cell line using different concentrations of GNP. Metabolic activity was evaluated using the MTT assay. Adapted with permission from Ref. [283]. Copyright 2010, American Chemical Society

Commonly, the neurotoxic effect of GNP has been evaluated only on neurons from different models through in vitro and in vivo assays, as described above. However, the CNS not only contains neurons but also glial cells, which are closely related to the normal functioning of the brain. There are different kinds of glia with specific functions, but in general, all of them participate in providing a key support system to the neurons [278, 279]. Thereby, the evaluation of GNP cytotoxicity both in neuronal models and in glia models allows for a general idea of the impact of GNP on the CNS. Only a few reports of the GNP effect on glia have been reported so far. Leite et al. [270] analyzed the effect of GNS-PEG on a 3D neural model to precisely determine the cytotoxicity that mimics the in vivo physiological conditions. This novel approach demonstrated that brainspheres were not affected by the different GNP used. Similar results were observed using GNS-PEG in either a human astrocyte cell line or primary cultures [275, 280]. This kind of information is key because astrocytes participate in maintaining adequate levels of some neurotransmitters, support the synaptic connection, and consolidate the learning and memory cognitive process [281]. Another relevant type of cell are microglia, which are the immune cells of the brain. Their activation or death could lead to the generation of neurodegenerative diseases or the loss of homeostasis. GNS with different coatings exhibited a non-cytotoxic effect in different microglial cell lines or primary cultures (Fig. 11) [267, 275, 277, 282, 283] as well as in in vivo assays analyzing either astrocytes or microglia [273, 284]. A similar non-cytotoxic effect was observed in GNCls with different coatings in in vitro assays for astrocytes or microglia [268, 269, 285–287]. Thus, the cytotoxic effect exhibited by GNP highly depends on the coating used, and modulating this parameter allows decreasing or avoiding these side effects in order to design and obtain safer GNP for potential therapies.

### Perspectives on strategies to enhance the analysis of GNP effect: 3D culture and the microfluidic model

GNP are potential tools to carry drugs and treat brain disorders. It is already known that GNP can be evaluated using in vitro cells in 2D cell cultures, which do not resemble the complexity of the in vivo nanoparticle-cell interaction. In this sense, dynamic methodologies highlight as interesting tools to introduce different variables that normally are not considered in static methods, mainly because a dynamic environment produces shear stress, changing the composition and structure of the biomolecules when they interact with nanoparticles [288–291]. In this sense, microfluidic (MF) has several advantages over conventional methods by incorporating fluid flow and mechanical forces, bringing a step closer to mimicking the in vivo microenvironment, being an in vivo-like method for studying nano-bio interfaces [292–295], for example, emulating the physiological conditions when administering nanoparticles into the bloodstream to reach different targets. In fact, MF corresponds to one of the pioneering techniques to analyze and study hydrodynamic regimes at small scales. This technique utilizes microscale channels to manipulate fluids at the nanoliter scale and suspend objects in a controlled manner [296], and it has rapidly progressed over the past decade, improving from basic devices to large-scale two-dimensional integration of components, 3D architectures, and nonlinear autoregulatory systems [297].

Currently, one of the future challenges is to obtain 3D in vitro nervous system models because the 2D cell cultures of neurite networks and in vitro brain cell architecture is limited and does not fully represent the function and complexity of the brain, especially during development and neuronal plasticity. The primary challenge in investigating drug delivery or any kind of molecule in 3D brain models lies in the complex and non-repetitive 3D structure of the brain, as well as its complex integrated circuits. In contrast, the current 2D cell culture models lack the ability to accurately replicate the complexity of the brain. At he moment, neuronal networks arranged in sophisticated 3D matrices or optimum combinations of different cells represent the inception of a groundbreaking generation that has the potential to revolutionize future results [298, 299]. Now, the field of bioengineering is striving to emulate the physiological systems with utmost accuracy to study the administration of nanoparticles. Accordingly, 3D in vitro models are the latest and most advanced method in this field. They provide the ability to replicate the physiological conditions seen in vivo [298, 300], resulting in improved cell survival, differentiation, and more accurate reproduction of electrical activity [270]. Lab-on-a-chip (LOC) has been used for neurobiology applications due to the ability of microfluidic channels to approximate the size and flow conditions found in in vivo capillaries, the so called in vivo-like flow. Therefore, studying the delivery of nanoparticles or their impact on specific biological systems is challenging due to dramatic variations in their efficacy across 2D and 3D culture systems [301].

**Fig. 12:**
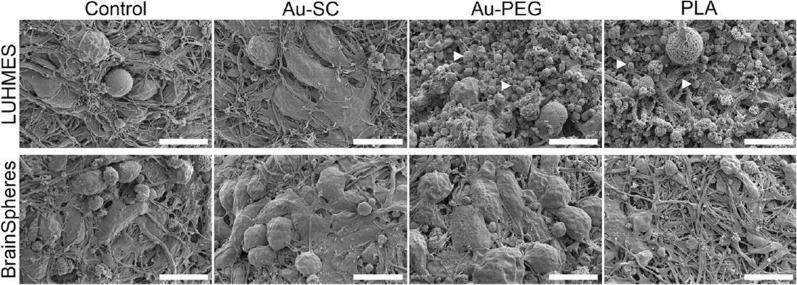
Morphology of 3D LUHMES and BrainSpheres exposed to Au-SC (6 μg/mL), Au-PEG (20 μg/mL) and PLA-NP (20 μg/mL) for 72 h. The control represents untreated spheroids. White arrowheads indicate cell debris. Scale bars: 10 μm. Adapted with permission from Ref. [270]. Copyright 2019, Springer Nature

**Fig. 13:**
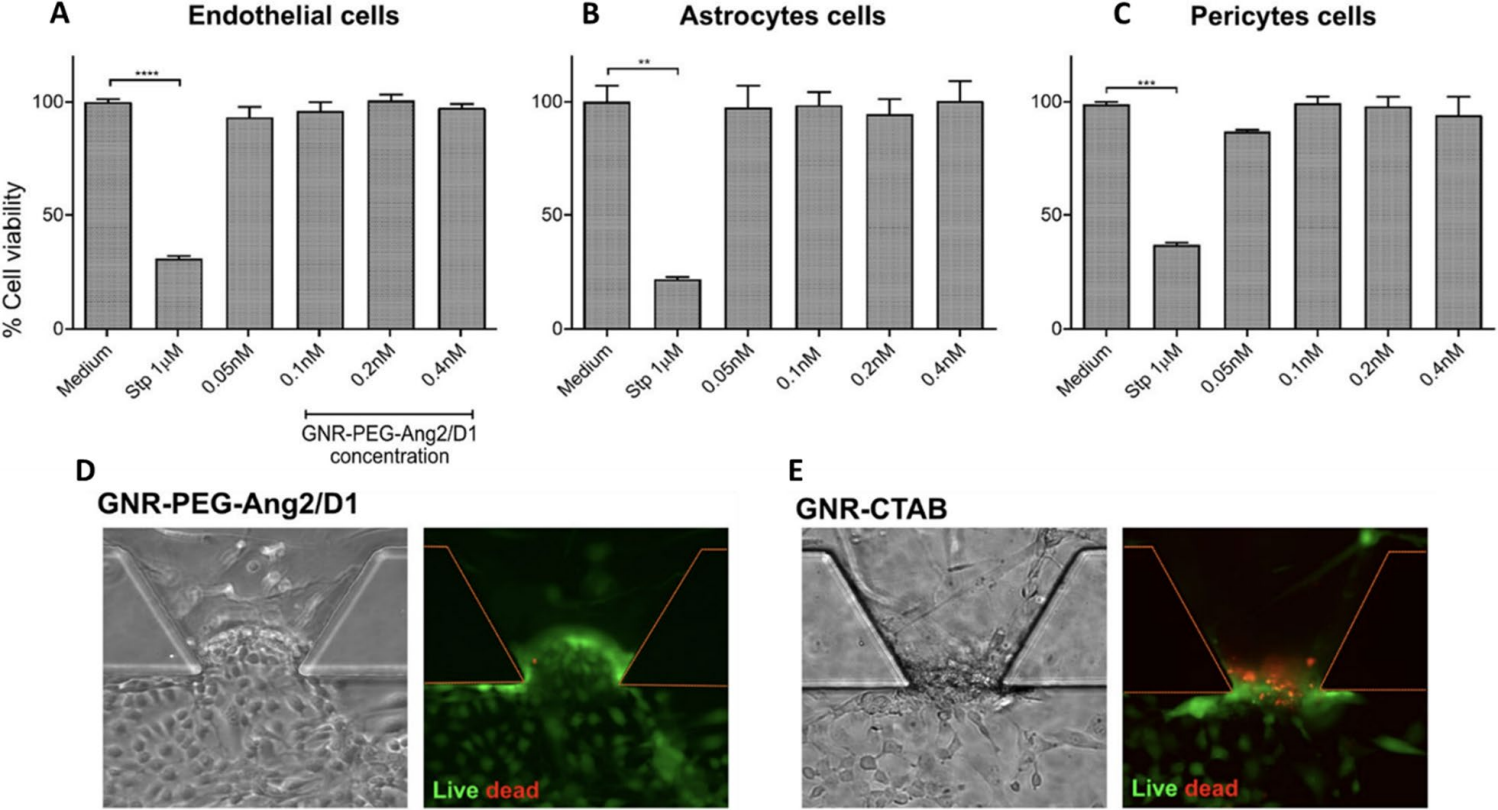
Cytotoxic effect of GNR-PEG-Ang2/D1 on cells that constitute the BBB at three different concentrations. A-C, cytotoxic effect of the nanosystem on each cell line, evaluated separately. D and E, GNR-PEG-Ang2/D1 analyzed on the endothelial barrier in the BBB-on-a-chip. GNR-CTAB was used as a control. Adapted with permission from Ref. [292]. Copyright, 2023 Springer Nature

In fact, there are microfluidic devices that simulate a complex functional and anatomical structure composed of endothelial cells and their BBB, forming tight junctions and constructing a 3D brain spheroid model to study the effect of GNP. Nevertheless, there are few publications describing these models to study the use of GNP [293, 302]. For example, Smirnova et al*.* [270] studied the effect of GNP functionalized with citrate and PEG using two different human 3D CNS in vitro models. In this work, they fabricated a 3D Lund human mesencephalic spheroid and a human iPSC-derived brain spheroid, called BrainSphere. The BrainSpheres is a multicellular 3D brain model composed of neurons, astrocytes, and oligodendrocytes, forming a complex multicellular model (Fig. 12). The comparison between single 3D models and a complex multicellular 3D model was relevant in the study of nanoparticle uptake, morphological and molecular alterations, and cytotoxicity, among others. The results indicated that GNP and their application in the 3D brain spheroid models are well suited to characterize the neurotoxicity of nanoparticles, and that the mixed populations of neural cells within the model allow the presence of glial cells (which participate in brain support). Therefore, GNP neurotoxicity decreased in comparison with the single 3D model, enhancing the comprehensiveness of this study in capturing physiological conditions. In this sense, a similar result was obtained by Palma-Florez et al. [292]. The authors mimicked the BBB with human astrocytes, pericytes and endothelial cells in a microfluidic device and analyzed a gold nanosystem, GNR-PEG-D1/Ang2, determining its non-cytotoxic effect on the tri-culture and the enhancement of nanosystem permeability (Fig. 13).

Likewise, it is important to mention that one of the key aspects of most protein folding or aggregation studies, as well as studies performed in the presence of inhibitory agents or GNP, are performed in bulk assays. This implies that the degree of freedom possessed by proteins or peptides is greater, so it is uncertain whether these studies truly reflect the behavior of a protein in living organisms [303]. Indeed, various studies have shown that the use of confined environments alters the kinetics and thermodynamics of protein–protein interactions [304], even promoting the appearance of β-secondary structures [305]. Additionally, since in vivo protein folding occurs mainly under rigid confinement characteristics, studies with models in confined spaces should be included in in vitro studies, constituting an interesting and challenging approach to replicate a typical scenario of the in vivo aggregation process [306–308]. In this sense, obtaining this information is mandatory because, bulk studies have indicated that the modification of the stirring speeds completely alters the aggregation process and morphology of the Aβ42 aggregates. In the same way, studies using microfluidics have shown that modifying the flow inside the microchannels together through a spatial confinement alters the morphology of amyloid aggregates [309, 310]. Furthermore, the confinement added to the low retention times achieved in the microchannels takes advantage of the initial stages of the amyloid aggregation process [311, 312], which are key steps directly related to the generation of neurotoxic oligomeric species of amyloid aggregates. Thus, the use of microfluidics emerges as a powerful methodology to study the aggregation process of amyloids in the presence of GNP, as well as their effect on the generation of toxic oligomeric species of amyloids. To date, no studies have used microfluidics to investigate the effect of functionalized GNP on the amyloid aggregation process in a confined space using, Tau or α-syn amyloid protein in a dynamic regime. Therefore, for the purpose of future studies, MF research has taken a keen interest in investigating this particular field of study due to the limited exploration of the aggregation process within microfluidic devices.

## Conclusions

Plasmonic GNP have gained interest in the biomedical field due to their high versatility and optical and electronic properties. This review highlighted their application in the development of diagnostic tools for PD and AD and described how the selectivity and sensitivity of current methods have been improved. In this sense, their use as delivery therapeutic agents or in vivo imaging has been proposed for the therapy and diagnosis of neurodegenerative diseases, taking advantage of the plasmonic properties of these nanomaterials. However, the research on GNP and their risk factors, neurotoxicity, targeting, selectivity, and sensitivity in the early stages of these diseases in preclinical models is incipient and remains a challenge. Furthermore, the investigation of the interactions between nanoparticles and biological barriers, as well as cellular responses, plays a critical role in developing preclinical and clinical applications of nanomaterials. For these reasons, cutting-edge methodologies such as 3D cultures and microfluidics have emerged as significant innovations by mimicking in vivo biological conditions, thus overcoming the limitations of bulk assays, and providing valuable and representative data for characterizing nanomaterials in relevant biological contexts. Furthermore, using microfluidics and "organ-on-a-chip" platforms highlights the emulation of physiological conditions to reduce the need for animal experimentation and contribute to understanding how GNP can interact with cells and cross the BBB. These advanced approaches are an essential step towards a deeper understanding of nanomaterial-cell interactions and the accurate assessment of their impact on the biological environment. In summary, the use of GNP in the diagnosis, targeting, therapy, and monitoring of neurodegenerative diseases represents an exciting and promising field in biomedical research. Although there are challenges ahead, such as safety, potential intranasal administration, the capping with biodegradable materials, or GNP biodistribution, the positive impact of these nanotools on the quality of patients’ lives and the advance in understanding these complex disorders is undeniable. It is crucial to maintain the collaboration between researchers and experts in various disciplines to make progress in this exciting field and potentially revolutionize the treatment of neurological diseases.

## Data Availability

Not applicable.

## Abbreviations

AD: Alzheimer’s disease
BBB: Blood–brain barrier
CNS: Central nervous system
GNP: Gold nanoparticles
NFTs: Neurofibrillary tangles
PD: Parkinson’s disease
P-Tau: Hyperphosphorylated Tau
T-Tau: Total Tau
α-syn: α-Synuclein
GNS: Gold nanospheres
GNR: Gold nanorods
GNPr: Gold nanoprisms
GNSts: Gold nanostars
GNCls: Gold nanoclusters
PEG: Polyethylene glycol
GNCs: Gold nanocomposites
MPTP: 1-Methyl-4-phenyl-1,2,3,6-tetrahydropyridine
ThT: Thioflavin-based kinetic assay
MNPs: Magnetic nanoparticles
LOD: Limit od detection
NGF: Nerve growth factor
MF: Microfluidics
